# Supplementary material for: Improving Child Neurology Residents' Communication Skills Through Objective Structured Clinical Exams
Source: MedEdPORTAL. 2021 Mar 4;17:11120. doi: 10.15766/mep_2374-8265.11120 (PMC7970633; doi:10.15766/mep_2374-8265.11120)
Supplement: Supplementary file 1 — Acute Stroke Scenario.docxMedical Error Scenario.docxStaring Spells Scenario.docxTourette Scenario.docxMigraine Scenario.docxDevelopmental Delay Scenario.docxDeath by Neurologic Criteria Scenario.docxPsychogenic Nonepileptic Events Scenario.docxNeonatal Hypoxic Ischemic Encephalopathy Scenario.docxFaculty & SP Assessment Form.docxLearner Self-Assessment Form.docxPost-OSCE Survey.docx [file mep_2374-8265.11120-s001.zip › A. Acute Stroke Scenario.docx]

**Child Neuro OSCE Case 1: Acute Stroke (Jackson)**

Date written: 10/7/2019

Primary Case Author: Margie Ream

Secondary Case Author: Dara VF Albert and Pedro Weisleder

Standardized Patient Educator: Todd Lash

Name of Case: Acute Stroke

Name of educational and or assessment activity: Gap-Kalamazoo Communication Skills Assessment Form, with modifications

Patient Name: Jackson, parents – Jenny and Sam

Chief Complaint: acute stroke in the cardiac intensive care unit (CTICU)

Most likely Diagnosis and Differential with rationale from history and/or physical exam: An acute left middle cerebral artery infarct is identified.

Challenge question: The challenge of this case is in counseling a family on a situation in which uncertainty and clinical equipoise exists.

Domains: Check all that apply

X Professionalism

X Communication and Interpersonal skills

- Medical History
- Physical exam

X Shared Decision Making

X Patient Education

- Clinical Reasoning
- Documentation
- Handoff
- Presentation
- Other:

Type and level of learner: pediatric and adult neurology residents (post-graduate years 2-5)

Case Objectives: please list specific objectives for each of the domains you have checked above:

1. Provide counseling to family regarding risks and benefits of stroke treatment.

2. Help family understand that not changing treatment immediately is the best course of action.

3. Demonstrate communication skills when there is not an immediate intervention.

| SETTING: | Inpatient in the Cardiac intensive care unit (CTICU) |
| --- | --- |
| PATIENT PROFILE: | |
| Age range | Patient is 6 months old, parents are both 40 years old |
| Religious/spiritual background | All may be used |
| Sex (e.g., male, female, intersex, transwoman, transman) | All may be used |
| Sexual Orientation (e.g., heterosexual, lesbian, gay, bisexual, pansexual, queer, asexual) | All may be used |
| Gender expression (e.g., man, woman, gender queer) | All may be used |
| Race/ethnicity: | All may be used |
| Physical description (e.g., BMI, height range) | All may be used |
| Physical limitations | All may be used |
| Patient appearance (e.g., disheveled, hospital gown, business casual, casual) | All may be used |
| Moulage + location (e.g., none, bruises, scars, body piercing, tattoos) | All may be used |
| Affect (e.g., pleasant, cooperative) | Anxious, uncomfortable with medical environment. |
| Family group (e.g., who is family, who they live with) | All may be used |
| Education | Technical school |
| Level of health literacy | Modest |
| Employment, if any - present and past, noting any current stresses | Both parents are employed. Dad is electrician. Mom is a part time secretary at a local veterinary office. |
| Home/homeless - type of dwelling, number of stories, owned or rented | All may be used |
| Financial situation- any current stresses | All may be used |
| Insurance Status (e.g., un/under/insured, public/private, HMO/PPO) | All may be used |
| Habits (i.e., diet, exercise, caffeine, smoking, alcohol, drugs) | All may be used |
| Activities (i.e., hobbies, sports, clubs, friends) | All may be used |
| Typical day - what is the usual daily routine | All may be used |

| CASE INFORMATION | |
| --- | --- |
| Chief Concern: | Seizure, arm weakness |
| Case Summary: | The resident is discussing treatment options for Jackson, a 6-month-old 5 kg infant with congenital heart disease. The patient is dependent on LVAD until a donor becomes available for a heart transplant. New onset seizures early that morning prompted a CT that showed an acute L MCA stroke. The patient has been on heparin and aspirin to prevent thrombosis. The resident must discuss the risks and benefits of treatment options in the setting of acute stroke in the CTICU. Family is worried because brain injury, either from hemorrhage or stroke, may impact the patient’s candidacy for a heart transplant. Hematology has deferred to neurology regarding the timing of starting additional antiplatelet therapy. Neurosurgery said the patient is too small for thrombectomy and we do not know exact timing of the stroke. The literature suggests that the greatest risk for hemorrhagic conversion is in the first 3 days of the stroke. Because there is not a clear guideline for how to proceed in this situation, the resident must present the options of starting additional platelet therapy now vs waiting 3 days including the risks and benefits. |
|  | |
| THE PATIENT STORY: | You are both older parents who tried for many years to have a second child. Your older child was 15 when this baby was born. A baby was finally conceived and was diagnosed prenatally with a congenital heart anomaly. You feel very anxious and protective of the baby you wanted so badly.  You are very anxious that she could have another stroke and had been told previously that heart transplants are given to patients who are expected to have a good neurologic outcome. You are very worried that this seizure and stroke will affect heart transplant candidacy, especially if she has another stroke. You thought that the medications to prevent strokes were supposed to work and don’t understand why this happened, but you do not want her to have any more strokes. You want to do everything possible to maintain her heart transplant candidacy. |
| HISTORY OF PRESENT ILLNESS:  The baby has abnormal coronary arteries leading to heart failure. She has been in the CTICU for 2 months with an LVAD awaiting a heart transplant. Her course has been uncomplicated until now. She has been on heparin and aspirin. Early this morning she had a brief seizure consisting of right arm jerking. The seizure prompted a CT which showed large left MCA acute infarct. She is now back to baseline except for subtle right arm weakness | |
|  | |
| REVIEW OF SYSTEMS: N/A | |
|  | |
| Past medical history |  |
| Medication allergies (Name and reaction) | NKDA |
| Environmental allergies (Name and reaction) | None |
| Illnesses | None |
| Vaccinations | Up to date |
| Surgeries | LVAD |
| Accidents/ injuries/ trauma | None |
| Hospitalization | Congenital heart disease, patient has been hospitalized for 2 months in the CTICU |
|  | |
| Inclusive sexual and reproductive history | |
| Sexual practices  Sexual partners  Protection: Use of safer sex practices  Use of birth control if appropriate  Risk of intimate partner violence | N/A |
| Ob/GYN HISTORY | N/A |
| Medications | Prescription/dose/reason  Over the counter/dose/reason  Herbs/supplements/dose/reason  Other: |
| Immunizations | - Tetanus - Flu - Hepatitis - Pneumovax - HPV   X Other – up to date for prior to 6 months |
| Tobacco products:   - Cigarettes - Cigar - Pipe - Chew - E-cigarettes | X Never   - Past- year started/year quit - Current   - Quantity   - # of years |
| Alcohol   - Beer - Wine - Liquor - Other | X Never   - Past- year started/year quit - Current   - Quantity   - # of years |
| Drugs   - Weed - Cocaine - Heroin - Meth - Other - IV - Inhalants - Other | X Never   - Past- year started/year quit - Current   - Quantity - # of years |
| Diet (describe) | N/A |
| Exercise (describe) | N/A |
| List any other important social history or information important to this case | N/A |
| Family history |  |
| Mother, Father, Siblings, Grandparents, and other significant findings. | Jackson’s older sister is 15 years old and healthy  Dad’s brother had a hemorrhagic stroke due to cocaine use.  Dad’s father died from a heart attack at 55 years old.  Mom’s mother had a L MCA stroke at 80 years old and was “trapped in her body, not able to talk or walk” for 6 years before she died in a nursing home. Had 2 miscarriages and one healthy baby (the child’s mother). |
| Physical Exam-  *Residents were not asked to complete a neurological exam.* | |
| PHYSICAL EXAM FINDINGS | None |
|  |  |
| DIAGNOSIS AND DIFFERENTIAL | Diagnosis is known to the learners |
|  |  |
| MANAGEMENT OR DIAGNOSTIC PLAN | Because there is not a clear guideline for how to proceed in this situation, the resident must present the options of starting additional platelet therapy now vs waiting 3 days including the risks and benefits. |
| PROFESSIONALISM ISSUES OR CHALLENGES: | Demonstrate communication skills when there is not an immediate intervention. |

**Jackson Door Instructions**

You are about to talk with the family of a 6-month-old infant with congenital heart disease who is dependent on an Left Ventricular Assist Device (LVAD) until she can receive heart transplant. New onset seizures early this morning prompted a CT that showed an acute left Middle Cerebral Artery (MCA) stroke. The patient has been on heparin and aspirin to prevent thrombosis. The patient is not a candidate for acute intervention (too small for thrombectomy, too high of a bleeding risk for thrombolytic therapy and we don’t have exact timing of the stroke). There is clinical equipoise as to the best next step in her management. Due to the large territory stroke, increasing her antiplatelet therapy immediately carries a risk of hemorrhagic transformation. Delaying additional therapy carries the risk of additional infarction. After consultation with stroke experts the medical team’s consensus is to delay additional therapy for 3 days. As you enter the room, the parents understand that she has had a stroke and that this was a potential complication of the LVAD. They also understand that extensive neurologic injury could affect her transplant candidacy, so they are very interested in limiting additional injury.

You are meeting with Jackson’s parents to discuss the risks and benefits of treatment options in the setting of acute stroke for this patient, including the options to start additional therapy now versus waiting 3 days. Please also help the family understand that not changing therapy now is the safest option and answer any questions about the long-term effects of left MCA stroke.

*Please keep in mind that you will have 20 minutes to complete the discussion with the parents. Also, please remember that you will be given feedback on how you communicate, not the content of that discussion or your clinical knowledge.*
