# Supplementary material for: Improving Child Neurology Residents' Communication Skills Through Objective Structured Clinical Exams
Source: MedEdPORTAL. 2021 Mar 4;17:11120. doi: 10.15766/mep_2374-8265.11120 (PMC7970633; doi:10.15766/mep_2374-8265.11120)
Supplement: Supplementary file 1 — Acute Stroke Scenario.docxMedical Error Scenario.docxStaring Spells Scenario.docxTourette Scenario.docxMigraine Scenario.docxDevelopmental Delay Scenario.docxDeath by Neurologic Criteria Scenario.docxPsychogenic Nonepileptic Events Scenario.docxNeonatal Hypoxic Ischemic Encephalopathy Scenario.docxFaculty & SP Assessment Form.docxLearner Self-Assessment Form.docxPost-OSCE Survey.docx [file mep_2374-8265.11120-s001.zip › B. Medical Error Scenario.docx]

**Child Neuro OSCE Case 2: Disclosure of a medical error (Michael)**

Date Written: 9/4/2019

Primary Case Author: Dara VF Albert

Secondary Case Author: Margie Ream, Pedro Weisleder

Standardized Patient Educator: Todd Lash

Name of Case: Disclosure of a Medical Error

Name of educational and or assessment activity: Gap-Kalamazoo Communication Skills Assessment Form, with modifications

Patient Name: Michael

Chief Complaint: Admission for breakthrough seizures

Most likely Diagnosis and Differential with rationale from history and/or physical exam: The patient is a child with a known diagnosis of epilepsy admitted for breakthrough seizures. The patient then develops likely aseptic meningitis after erroneously receiving a dose of intravenous immunoglobulin (IVIG).

Challenge question: The resident must disclose that an error has occurred and the patient has received a medication with potential consequences.

Domains: Check all that apply

X Professionalism

X Communication and Interpersonal skills

- Medical History
- Physical exam
- Shared Decision Making

X Patient Education

- Clinical Reasoning
- Documentation
- Handoff
- Presentation
- Other:

Type and level of learner: pediatric and adult neurology residents (post-graduate years 2-5)

Case Objectives: please list specific objectives for each of the domains you have checked above:

1. Resident must disclose a medical error to a patient and their family.

2. Demonstrate communication skills in what could be an adversarial situation in which an error occurred that lead to patient harm.

3. Articulate the process for understanding how a medical error occurred without laying blame on anyone individual on the medical team.

| SETTING: | Inpatient neurology ward |
| --- | --- |
| PATIENT PROFILE: | |
| Age range | Patient is 10 years old, the parents are both in their 40s |
| Religious/spiritual background | All may be used |
| Sex (e.g., male, female, intersex, transwoman, transman) | All may be used |
| Sexual Orientation (e.g., heterosexual, lesbian, gay, bisexual, pansexual, queer, asexual) | All may be used |
| Gender expression (e.g., man, woman, gender queer) | All may be used |
| Race/ethnicity: | All may be used |
| Physical description (e.g., BMI, height range) | All may be used |
| Physical limitations | All may be used |
| Patient appearance (e.g., disheveled, hospital gown, business casual, casual) | All may be used |
| Moulage + location (e.g., none, bruises, scars, body piercing, tattoos) | None |
| Affect (e.g., pleasant, cooperative) | The parents are upset about the incorrect medication given to the child and are concerned about the potential consequences. |
| Family group (e.g., who is family, who they live with) | all may be used |
| Education | The child is in 4th grade, both parents attended some college |
| Level of health literacy | Moderate |
| Employment, if any - present and past, noting any current stresses | Both parents work in retail and are concerned about missing additional days of work due to an extended inpatient stay |
| Home/homeless - type of dwelling, number of stories, owned or rented | All may be used |
| Financial situation- any current stresses | Middle-income family |
| Insurance Status (e.g., un/under/insured, public/private, HMO/PPO) | All may be used |
| Habits (i.e., diet, exercise, caffeine, smoking, alcohol, drugs) | All may be used |
| Activities (i.e., hobbies, sports, clubs, friends) | All may be used |
| Typical day - what is the usual daily routine | All may be used |

| CASE INFORMATION | |
| --- | --- |
| Chief Concern: | Admission for breakthrough seizures |
| Additional Concerns: | This morning the patient is having low-grade fevers as well as a moderate headache |
|  | |
| THE PATIENT STORY: | You are the parents of Michael, a 10-year-old boy with well-controlled epilepsy. You are feeling somewhat guilty that he missed a few doses of medication and that triggered a prolonged seizure necessitating this hospital admission.  You are angry about the error and asks multiple times how this could have happened. In your anger, you bring up getting a lawyer and “suing the hospital”.  You are also worried about the consequences of receiving intravenous immunoglobulin (IVIG), specifically that he is now having symptoms and also could it make his seizures worse. If the resident brings up “aseptic meningitis” you should again get angry and worried that “meningitis can cause permanent brain damage”.  When you are told IVIg is a blood product, you raise concern for blood-borne illnesses such as hepatitis or HIV.  If the resident attempts to shift the blame to the nurse, you should get defensive of the nurse and say it was not her fault, “she was just following orders”.  If resident offers to contact patient visitor relations you should accept but continue to engage resident in the issue. If resident blames the intern, you can redirect anger and blame to the intern and question if he was being supervised appropriately and by whom. |
| HISTORY OF PRESENT ILLNESS:  Michael is a 10-year-old boy with history of well-controlled idiopathic generalized epilepsy, who is admitted to the neurology service for breakthrough seizures in the setting of missed doses of medication. The family had been on vacation and did not pack enough medicine to get through the week. He had a prolonged (30 minute) seizure the night they returned from vacation. After receiving a loading dose of medication, he had been resting comfortably with no further seizures overnight.  The next morning, Michael is erroneously given a therapy that was meant for another patient, IVIg, which is a blood product that carries risks.  There is another patient on the service who was admitted with Guillian-Barre syndrome, which requires IVIg treatment. The neurology resident accidentally advised the pediatric intern to order IVIg for “room 30”, which is Michael’s room but the medication was meant for “room 31”. The intern did not ask clarifying questions and entered the order as instructed. An inexperienced nurse gave Michael the IVIg despite lack of consent in the chart. Patient now has a headache and low-grade fever suggesting aseptic meningitis.  Resident needs to explain to Michael’s family that the error has occurred and review the risks of IVIg therapy. | |
|  | |
| REVIEW OF SYSTEMS: Significant positives and negatives | |
| headaches, low-grade fever, seizures | |
| Past medical history |  |
| Medication allergies (Name and reaction) | NKDA |
| Environmental allergies (Name and reaction) | None |
| Illnesses | Epilepsy, well-controlled on levetiracetam monotherapy |
| Vaccinations | Up to date |
| Surgeries | None |
| Accidents/ injuries/ trauma | None |
| Hospitalization | None prior to this current admission |
|  | |
| Inclusive sexual and reproductive history | |
| Sexual practices  Sexual partners  Protection: Use of safer sex practices  Use of birth control if appropriate  Risk of intimate partner violence | N/A |
| Ob/GYN HISTORY | N/A |
| Medications | levetiracetam |
| Immunizations | X up to date |
| Tobacco products:   - Cigarettes - Cigar - Pipe - Chew - E-cigarettes | X Never   - Past- year started/year quit - Current   - Quantity   - # of years |
| Alcohol   - Beer - Wine - Liquor - Other | X Never   - Past- year started/year quit - Current   - Quantity   - # of years |
| Drugs   - Weed - Cocaine - Heroin - Meth - Other - IV - Inhalants - Other | X Never   - Past- year started/year quit - Current   - Quantity - # of years |
| Diet (describe) | Typical American diet |
| Exercise (describe) | Active, plays soccer and baseball |
| List any other important social history or information important to this case | The child is in the 4th grade, doing well. Both parents work in retail and there is some concern about missing additional days due to hospitalization |
| Family history |  |
| Mother, Father, Siblings, Grandparents, and other significant findings. | Father had epilepsy as a child that he outgrew  5-year-old brother who is healthy  Mother has a history of anxiety |
| Physical Exam-  *Residents were not asked to complete a neurological exam.* | |
| PHYSICAL EXAM FINDINGS | None |
|  |  |
| DIAGNOSIS AND DIFFERENTIAL | Diagnosis is known to the learners |
|  |  |
| MANAGEMENT OR DIAGNOSTIC PLAN | The resident must disclose the medical error, answer the parent’s questions, and discuss next steps. |
| PROFESSIONALISM ISSUES OR CHALLENGES: | A child neurology resident is supervising a busy neurology service with many (10+) complex patients. A mix up occurs and a medication meant for one patient is given to the wrong patient. Although the error is not entirely the resident’s fault, he/she must disclose the error to the family in a compassionate way that is honest and attempts to re-establish the family’s trust in the medical team/institution. |

**Michael Door Instructions**

You are the senior resident on a busy inpatient Neurology consult service caring for 10 complex patients with neurological illness.

Michael is a 10-year-old boy with a history of well-controlled idiopathic generalized epilepsy and was admitted for an increase in seizures in the setting of missed doses of medication. The family had been on vacation and did not pack enough medicine to get through the week. He had a prolonged (30 minute) seizure the night they returned from vacation. After receiving a loading dose of medication, he had been resting comfortably with no further seizures overnight.

The next morning, Michael is erroneously given a therapy that was meant for another patient, intravenous immunoglobulin (IVIg). There is another patient on the service who was admitted with Guillian-Barre syndrome. You advised the pediatric intern to order IVIg for “room 30”, which is Michael’s room, but the medication was meant for “room 31”. The intern did not ask clarifying questions and entered the order as instructed. An inexperienced nurse gave Michael the IVIg despite lack of consent in the chart. Patient now has a headache and low-grade fever suggesting aseptic meningitis.

Michael's parents are waiting in the conference room to discuss this with you.

*Please keep in mind that you will have 20 minutes to complete the discussion with the parents. Also, please remember that you will be given feedback on how you communicate, not the content of that discussion or your clinical knowledge.*
