# Supplementary material for: Improving Child Neurology Residents' Communication Skills Through Objective Structured Clinical Exams
Source: MedEdPORTAL. 2021 Mar 4;17:11120. doi: 10.15766/mep_2374-8265.11120 (PMC7970633; doi:10.15766/mep_2374-8265.11120)
Supplement: Supplementary file 1 — Acute Stroke Scenario.docxMedical Error Scenario.docxStaring Spells Scenario.docxTourette Scenario.docxMigraine Scenario.docxDevelopmental Delay Scenario.docxDeath by Neurologic Criteria Scenario.docxPsychogenic Nonepileptic Events Scenario.docxNeonatal Hypoxic Ischemic Encephalopathy Scenario.docxFaculty & SP Assessment Form.docxLearner Self-Assessment Form.docxPost-OSCE Survey.docx [file mep_2374-8265.11120-s001.zip › C. Staring Spells Scenario.docx]

**Child Neuro OSCE Case 4: Staring spells (Eli)**

Date Written: 12/8/2017

Primary Case Author: Dara VF Albert

Secondary Case Author: Margie Ream, Pedro Weisleder

Standardized Patient Educator: Todd Lash

Name of Case: Staring Spells

Name of educational and or assessment activity: Gap-Kalamazoo Communication Skills Assessment Form, with modifications

Patient Name: Eli

Chief Complaint: staring spells

Most likely Diagnosis and Differential with rationale from history and/or physical exam: The most likely diagnosis in this scenario is childhood absence epilepsy.

Challenge question: The resident needs to utilize the clinical history a neurodiagnostic testing to arrive at a diagnosis and educate the parent about that diagnosis as well as treatment.

Domains: Check all that apply

- Professionalism

X Communication and Interpersonal skills

- Medical History
- Physical exam

X Shared Decision Making

X Patient Education

- Clinical Reasoning
- Documentation
- Handoff
- Presentation
- Other:

Type and level of learner: pediatric and adult neurology residents (post-graduate years 2-5)

Case Objectives: please list specific objectives for each of the domains you have checked above:

1. Resident to counsel the family regarding impression and plan based on the diagnosis using clear and effective communication.

2. Resident to use clinical history and neurodiagnostic testing to arrive at a diagnosis.

3. Resident to respond to and address the parent’s anxiety.

| SETTING: | outpatient Neurology Clinic |
| --- | --- |
| PATIENT PROFILE: | |
| Age range | The patient is 7 years old, the parent is 35 to 40 years old |
| Religious/spiritual background | All may be used |
| Sex (e.g., male, female, intersex, transwoman, transman) | All may be used |
| Sexual Orientation (e.g., heterosexual, lesbian, gay, bisexual, pansexual, queer, asexual) | All may be used |
| Gender expression (e.g., man, woman, gender queer) | All may be used |
| Race/ethnicity: | All may be used |
| Physical description (e.g., BMI, height range) | All may be used |
| Physical limitations | All may be used |
| Patient appearance (e.g., disheveled, hospital gown, business casual, casual) | All may be used |
| Moulage + location (e.g., none, bruises, scars, body piercing, tattoos) | None |
| Affect (e.g., pleasant, cooperative) | The parent is anxious and talkative with many questions, but should allow resident to complete the interview and counsel |
| Family group (e.g., who is family, who they live with) | All may be used |
| Education | The parent received a professional degree |
| Level of health literacy | Modest, health literacy does not match high level of non-medical education |
| Employment, if any - present and past, noting any current stresses | The parent is a high-achieving engineer |
| Home/homeless - type of dwelling, number of stories, owned or rented | All may be used |
| Financial situation- any current stresses | High middle class |
| Insurance Status (e.g., un/under/insured, public/private, HMO/PPO) | All may be used |
| Habits (i.e., diet, exercise, caffeine, smoking, alcohol, drugs) | All may be used |
| Activities (i.e., hobbies, sports, clubs, friends) | All may be used |
| Typical day - what is the usual daily routine | All may be used |

| CASE INFORMATION | |
| --- | --- |
| Chief Concern: | Staring spells |
| Additional Concerns: | The parent comes to clinic to discuss the results of an evaluation of staring spells in his/her 7-year-old child. The electroencephalogram (EEG) was done and it captured typical events that were correlated with 3 Hz spike-and-wave discharges congruent with absence seizures. The parent is asked to come back to clinic after the EEG to discuss the results. |
|  | |
| THE PATIENT STORY: | You are the parent of 7-year-old Eli who has been evaluated in Neurology Clinic. You are a highly analytical, data-driven, educated person. You believe these staring episodes you have been seeing are either nothing or could be Attention Deficit Disorder/Hyperactivity Disorder like his older sister. You are very surprised to hear the diagnosis of epilepsy and immediately become anxious given that your father “died from a seizure”.  Also, when you hear that Eli has been having seizures, you feel guilty that you “missed” something serious. Especially given that he recently broke his arm, you wonder if maybe he had a seizure and that caused him to fall from his bike. And, you have been “giving him a hard time” about his grades slipping this school year.  In addition, you are someone who “does not like medicine” and are resistant to the idea of “medicating your child”. You wonder about “natural” ways to treat the seizures. If the resident brings up treatment with medication, you should ask the following questions:   - Why do we need to treat with medications? - What are the side effects? - Can we treat this naturally, like with medical marijuana? I have heard that is being used to treat seizures |
| HISTORY OF PRESENT ILLNESS:  7-year-old Eli with staring spells for about 6 months noticed at home and at school. Episodes are brief behavioral arrest with staring and eyelid fluttering lasting no more than 15 seconds followed by return to baseline. No change in tone or color associated with the episodes. No abnormal movements of the body. No falls with the episodes. Sometimes he is staring off at the TV and we can’t get his attention by calling his name. He has also starred off while talking to me. Teachers complain that he is daydreaming a lot in school. His grades have slipped from As to Bs this year. He isn’t aware that he is staring off. Older sister has ADHD which was diagnosed at 7 years. She would stare off as well and now is better with medication. Mom thinks that is what is going on with Eli.  Mom will be surprised when epilepsy diagnosis is made and will have lots of questions.  - What causes the seizures?  - Will he outgrow the seizures?  - Are the seizures causing brain damage?  - Can the child die from the seizures?  - Do we need a brain MRI?   - Is this ADHD? - Will this affect his ability to learn in school? - Making a connection to mom’s dad who died of a stroke- could these seizures be caused by a stroke in Eli? - Does he need medicine? - What if medicine doesn’t help? - Will medicine change his behavior/personality- “I don’t want him to be a zombie” - Is there something I could have done to prevent this? - Why does he have seizures? Why did this happen? Where do seizures come from? Don’t we need an MRI? (if resident says its genetic- do we need to do genetic testing?) - Is there risk of brain damage? - Is there a chance he could die from this? - Will he outgrow these? - If this is benign, why treat? - Can he play sports? How much freedom should I allow him vs restricting him? | |
|  | |
| REVIEW OF SYSTEMS: Significant positives and negatives | |
| None | |
| Past medical history |  |
| Medication allergies (Name and reaction) | NKDA |
| Environmental allergies (Name and reaction) | None |
| Illnesses | Mild intermittent asthma diagnosed at 2 years of age |
| Vaccinations | Up to date |
| Surgeries | None |
| Accidents/ injuries/ trauma | About 6 months ago he fell from his bike and broke his arm |
| Hospitalization | None, has been to the ER for asthma exacerbations as well as when he broke his arm |
|  | |
| Inclusive sexual and reproductive history | |
| Sexual practices  Sexual partners  Protection: Use of safer sex practices  Use of birth control if appropriate  Risk of intimate partner violence | N/A |
| Ob/GYN HISTORY | N/A |
| Medications | Albuterol as needed, multivitamin |
| Immunizations | X up to date |
| Tobacco products:   - Cigarettes - Cigar - Pipe - Chew - E-cigarettes | X Never   - Past- year started/year quit - Current   - Quantity   - # of years |
| Alcohol   - Beer - Wine - Liquor - Other | X Never   - Past- year started/year quit - Current   - Quantity   - # of years |
| Drugs   - Weed - Cocaine - Heroin - Meth - Other - IV - Inhalants - Other | X Never   - Past- year started/year quit - Current   - Quantity - # of years |
| Diet (describe) | Typical American diet |
| Exercise (describe) | Active, plays soccer after school |
| List any other important social history or information important to this case | He is currently in the 2nd grade, grades have been slipping somewhat this year, coinciding with when the staring events started. |
| Family history |  |
| Mother, Father, Siblings, Grandparents, and other significant findings. | Parents are both healthy  9-year-old sister with Attention Deficit Disorder/Hyperactivity Disorder  Maternal grandfather had an ischemic stroke at 60 and developed post-stroke epilepsy. He died at 70 secondary to a brain bleed after a seizure (he fell and hit his head)  Maternal grandmother is healthy  Paternal grandmother has hypertension and anxiety  Paternal grandfather has diabetes |
|  |  |
| Physical Exam-  *Residents were not asked to complete a neurological exam.* | |
| PHYSICAL EXAM FINDINGS | None |
|  |  |
| DIAGNOSIS AND DIFFERENTIAL | Diagnosis is known to the learners |
|  |  |
| MANAGEMENT OR DIAGNOSTIC PLAN | The child most likely has childhood absence epilepsy. The residents need to discuss the diagnosis, answer questions and concerns from the parent. |
|  |  |
| PROFESSIONALISM ISSUES OR CHALLENGES: | Although this is a comment diagnosis, the parent is extremely anxious and overly analytical with underlying, unspoken guilt. |

**Eli Door Instructions**

The mother of a 7-year-old boy comes to the clinic to follow up on test results after he underwent an evaluation for staring spells. The mother described frequent, brief events of behavioral arrest with staring and unresponsiveness lasting 10-20 seconds. The patient is unaware that anything had occurred after an event. His teachers had also reported these events to the parents after they started noticing them at school. Based on the clinical history, you ordered a routine EEG and mom returns today to discuss the results. The EEG showed brief bursts of interictal generalized 3 Hz spike-and-wave discharges. In addition, typical clinical events were captured during hyperventilation that are correlated with 3 Hz spike-and-wave discharges congruent with absence seizures.

Please review these results and the clinical relevance with the mother as well as answer any questions she may have.

*Please keep in mind that you will have 20 minutes to complete the discussion with the mother. Also, please remember that you will be given feedback on how you communicate with the parent, not the content of that discussion or your clinical knowledge.*
