# Supplementary material for: Improving Child Neurology Residents' Communication Skills Through Objective Structured Clinical Exams
Source: MedEdPORTAL. 2021 Mar 4;17:11120. doi: 10.15766/mep_2374-8265.11120 (PMC7970633; doi:10.15766/mep_2374-8265.11120)
Supplement: Supplementary file 1 — Acute Stroke Scenario.docxMedical Error Scenario.docxStaring Spells Scenario.docxTourette Scenario.docxMigraine Scenario.docxDevelopmental Delay Scenario.docxDeath by Neurologic Criteria Scenario.docxPsychogenic Nonepileptic Events Scenario.docxNeonatal Hypoxic Ischemic Encephalopathy Scenario.docxFaculty & SP Assessment Form.docxLearner Self-Assessment Form.docxPost-OSCE Survey.docx [file mep_2374-8265.11120-s001.zip › D. Tourette Scenario.docx]

**Child Neuro OSCE Case 3: Tourette syndrome (Otis)**

Date Written: 9/4/2019

Primary Case Author: Pedro Weisleder

Secondary Case Author: Margie Ream, Dara VF Albert

Standardized Patient Educator: Todd Lash

Name of Case: Tourette syndrome

Name of educational and or assessment activity: Gap-Kalamazoo Communication Skills Assessment Form, with modifications

Patient Name: Otis

Chief Complaint: Tics

Most likely Diagnosis and Differential with rationale from history and/or physical exam: The patient meets criteria for a diagnosis of Tourette syndrome.

Challenge question:

Domains: Check all that apply

X Professionalism

X Communication and Interpersonal skills

- Medical History
- Physical exam
- Shared Decision Making

X Patient Education

- Clinical Reasoning
- Documentation
- Handoff
- Presentation
- Other:

Type and level of learner: pediatric and adult neurology residents (post-graduate years 2-5)

Case Objectives: please list specific objectives for each of the domains you have checked above:

1. Explain to the patient and his family what Tourette syndrome is.

2. Address the patient’s anxiety as it relates to having a poorly understood medical condition. A condition that in the era of social media, is stigmatized by the most extreme examples.

3. Suggest strategies to try to overcome the components of #2.

| SETTING: | outpatient Neurology Clinic |
| --- | --- |
| PATIENT PROFILE: Adolescent with Tics  (the case was written for a male adolescent patient, however pronouns could be interchangeable and the name could be changed if a female patient is preferred) | |
| Age range | The patient is 15 years old, the parent is 45 years old (for this case a young-appearing standardized patient can be recruited to portray the adolescent) |
| Religious/spiritual background | All may be used |
| Sex (e.g., male, female, intersex, transwoman, transman) | All may be used |
| Sexual Orientation (e.g., heterosexual, lesbian, gay, bisexual, pansexual, queer, asexual) | All may be used |
| Gender expression (e.g., man, woman, gender queer) | All may be used |
| Race/ethnicity: | All may be used |
| Physical description (e.g., BMI, height range) | All may be used |
| Physical limitations | All may be used |
| Patient appearance (e.g., disheveled, hospital gown, business casual, casual) | All may be used |
| Moulage + location (e.g., none, bruises, scars, body piercing, tattoos) | None |
| Affect (e.g., pleasant, cooperative) | The patient seems anxious. He has a difficult time seating still. Occasionally, he/she clears his/her throat or has facial twitches. He is embarrassed by what his parent is telling the doctor.  The parent is pleasant but concerned about the son and does most of the talking. |
| Family group (e.g., who is family, who they live with) | All may be used |
| Education | The patient is a freshman in high school, the parent is a college graduate |
| Level of health literacy | Moderate |
| Employment, if any - present and past, noting any current stresses | The patient is in high school, both parents are working professionals, one in business and the other in IT.  Both parents are dedicated to their careers. That said, mother seems to shoulder most of the work of taking care of the children. She is the one that takes the children to their extracurricular activities. Parents have good jobs, they are middle class. |
| Home/homeless - type of dwelling, number of stories, owned or rented | All may be used |
| Financial situation- any current stresses | All may be used |
| Insurance Status (e.g., un/under/insured, public/private, HMO/PPO) | All may be used |
| Habits (i.e., diet, exercise, caffeine, smoking, alcohol, drugs) | All may be used |
| Activities (i.e., hobbies, sports, clubs, friends) | All may be used |
| Typical day - what is the usual daily routine | All may be used |

| CASE INFORMATION | |
| --- | --- |
| Chief Concern: | Tics |
| Additional Concerns: | Patient and parent want to know if he/she has Tourette syndrome. There are also underlying social/peer and academic concerns related to the tics. |
|  | |
| THE PATIENT STORY: | As the adolescent, you are nervous and embarrassed by your tics as well as your parents’ concerns. You prefer that your parents “just leave you alone”. You have experienced peers making fun of your tics. You are withdrawn and give poor eye contact during the encounter, and only open up to the resident after they actively try to engage you.  As the parent, you are concerned about your child. He/She is often quiet at home and does not share his/her feelings with you. You are concerned he/she may be struggling emotionally more than he/she is letting on. |
| HISTORY OF PRESENT ILLNESS:  The patient is a 15-year-old adolescent referred to the neurology clinic for evaluation of motor and vocal tics that have been present for at least 5 years. The adolescent’s parent wants to know if the boy he/she has Tourette syndrome because they believe the aldolescent has tics. They are also concerned that the adolescent could have Parkinson disease or “something more progressive/degenerative” like his/her maternal grandmother.  The symptoms began around age 10 years. The adolescent started blinking often, for that reason he/she was taken to see an optometrist who said the vision was fine. A few months later the adolescent began clearing his/her throat, so he/she was referred to an allergist who said that there was no evidence of an allergic condition.  At one time or another, the adolescent has exhibited the following: frequent blinking, shoulder shrugging, head turning. He/she has also cleared his throat even if he/she does not have a cold, he/she has made a barking sound, and he/she has repeated the last words other people say.  Around age 12, the teachers reported that the adolescent could not stay seated. He/she interrupted other kids in class, and he/she could not complete the school assignments on time. For that reason, the adolescent was prescribed Ritalin. The medication “seems to help.” But he/she can still get in trouble at school.  Parents think that it is unusual that a teenager is “obsessed” with order. He/she likes his/her clothes arranged by color and gets upset if someone alters the order. His/her older brother keeps a mess in his room. Parents would like both to be “in the middle.” | |
|  | |
| REVIEW OF SYSTEMS: Significant positives and negatives | |
| “obsessed” with order, picky eater | |
| Past medical history |  |
| Medication allergies (Name and reaction) | NKDA |
| Environmental allergies (Name and reaction) | None |
| Illnesses | None |
| Vaccinations | up to date |
| Surgeries | None |
| Accidents/ injuries/ trauma | None |
| Hospitalization | None |
|  | |
| Inclusive sexual and reproductive history | |
| Sexual practices | N/A |
| Ob/GYN HISTORY | N/A |
| Medications |  |
| Immunizations | X up to date |
| Tobacco products:   - Cigarettes - Cigar - Pipe - Chew - E-cigarettes | X Never   - Past- year started/year quit - Current   - Quantity   - # of years |
| Alcohol   - Beer - Wine - Liquor - Other | X Never   - Past- year started/year quit - Current   - Quantity   - # of years |
| Drugs   - Weed - Cocaine - Heroin - Meth - Other - IV - Inhalants - Other | X Never   - Past- year started/year quit - Current   - Quantity - # of years |
| Diet (describe) | Picky eater, but dislikes food with certain textures such as mashed potatoes |
| Exercise (describe) | Plays in a soccer league |
| List any other important social history or information important to this case | Adolescent is quiet and reserved, but embarrassed about his/her tics and has experienced being made fun of by peers |
| Family history |  |
| Mother, Father, Siblings, Grandparents, and other significant findings. | Mother reports being “bipolar”  Older brother with Attention Deficit Disorder/Hyperactivity Disorder  Father has well-controlled diabetes  Maternal grandfather is deceased from sudden myocardial infarction  Maternal grandmother has Parkinson's disease  History is not known about paternal grandparents |
|  |  |
| Physical Exam-  *Residents were not asked to complete a neurological exam.* | |
| PHYSICAL EXAM FINDINGS | During the visit the adolescent frequently made a humming sound, clear his/her throat or repeatedly blink. Occasionally, the adolescent would forcefully turn his/her head to the right. Otherwise, he/she is very quiet with poor eye contact until actively engaged by resident. |
|  |  |
| DIAGNOSIS AND DIFFERENTIAL | Diagnosis is known to the learners |
|  |  |
| MANAGEMENT OR DIAGNOSTIC PLAN | The adolescent exhibits typical symptoms of Tourette syndrome and the resident should provide this diagnosis. He/she also exhibits some symptoms of anxiety as well as “OCD-like” behaviors |
|  |  |
| PROFESSIONALISM ISSUES OR CHALLENGES: | The resident should attempt to connect with the withdrawn teenager and elicit his underlying anxiety and social concerns related to his tics. |

**Otis Door Instructions**

You are asked to evaluate Otis, a 15-year-old young man who displays motor and vocal tics that have been present for at least 5 years. Initially, the patient was blinking frequently. For that reason, he was taken to see an optometrist who said the vision was fine. A few months later the child began clearing his throat, so he was referred to an allergist who said that there was no evidence of an allergic condition. At times, the symptoms have happened multiple times a day. In other instances, there appears to be a lull where the symptoms have hardly been present. The child has been struggling in school as he is unable to sit still. Parents mentioned that the young man likes to arrange his clothes by color and becomes very upset if the order is altered.

You are expected to explain to the patient and his family what Tourette syndrome is as well as address the patient’s anxiety as it relates to having a poorly understood medical condition.

*Please keep in mind that you will have 20 minutes to complete the discussion with the patient and parents. Also, please remember that you will be given feedback on how you communicate, not the content of that discussion or your clinical knowledge.*
