# Supplementary material for: Improving Child Neurology Residents' Communication Skills Through Objective Structured Clinical Exams
Source: MedEdPORTAL. 2021 Mar 4;17:11120. doi: 10.15766/mep_2374-8265.11120 (PMC7970633; doi:10.15766/mep_2374-8265.11120)
Supplement: Supplementary file 1 — Acute Stroke Scenario.docxMedical Error Scenario.docxStaring Spells Scenario.docxTourette Scenario.docxMigraine Scenario.docxDevelopmental Delay Scenario.docxDeath by Neurologic Criteria Scenario.docxPsychogenic Nonepileptic Events Scenario.docxNeonatal Hypoxic Ischemic Encephalopathy Scenario.docxFaculty & SP Assessment Form.docxLearner Self-Assessment Form.docxPost-OSCE Survey.docx [file mep_2374-8265.11120-s001.zip › E. Migraine Scenario.docx]

**Child Neuro OSCE Case 5: Migraine headaches (Veronica)**

Date Written: 1/27/2018

Primary Case Author: Pedro Weisleder

Secondary Case Author: Margie Ream, Dara VF Albert

Standardized Patient Educator: Todd Lash

Name of Case: Migraines

Name of educational and or assessment activity: Gap-Kalamazoo Communication Skills Assessment Form, with modifications

Patient Name: Veronica

Chief Complaint: Headaches

Most likely Diagnosis and Differential with rationale from history and/or physical exam: The patient most likely is suffering from migraine headaches that are chronic in nature.

Challenge question: The resident must reassure the patient and her mother regarding they are concerned that headaches could be caused by something more sinister.

Domains: Check all that apply

X Professionalism

X Communication and Interpersonal skills

- Medical History
- Physical exam

X Shared Decision Making

X Patient Education

- Clinical Reasoning
- Documentation
- Handoff
- Presentation
- Other:

Type and level of learner: pediatric and adult neurology residents (post-graduate years 2-5)

Case Objectives: please list specific objectives for each of the domains you have checked above:

1. Explain to the family why a test is not indicated.

2. Demonstrate communication skills in what could be an adversarial situation.

3. Demonstrate empathy and seek to understand the parent/family prospective regarding management

| SETTING: | outpatient Neurology Clinic |
| --- | --- |
| PATIENT PROFILE: | |
| Age range | The patient is 16 years old, the parent is mid-40s |
| Religious/spiritual background | All may be used |
| Sex (e.g., male, female, intersex, transwoman, transman) | All may be used |
| Sexual Orientation (e.g., heterosexual, lesbian, gay, bisexual, pansexual, queer, asexual) | All may be used |
| Gender expression (e.g., man, woman, gender queer) | All may be used |
| Race/ethnicity: | All may be used |
| Physical description (e.g., BMI, height range) | All may be used |
| Physical limitations | All may be used |
| Patient appearance (e.g., disheveled, hospital gown, business casual, casual) | All may be used |
| Moulage + location (e.g., none, bruises, scars, body piercing, tattoos) | None |
| Affect (e.g., pleasant, cooperative) | The mother is irritable as the doctor does not want to order the tests that she wants, she gets angry and dominates the conversation. The adolescent is quiet, withdrawn and makes little eye contact. Does not speak unless directly addressed |
| Family group (e.g., who is family, who they live with) | All may be used |
| Education | The patient is in high school, the parent graduated from high school and did not achieve higher levels of education |
| Level of health literacy | Low to modest |
| Employment, if any - present and past, noting any current stresses | The parent has a low-paying clerical job |
| Home/homeless - type of dwelling, number of stories, owned or rented | All may be used |
| Financial situation- any current stresses | Single parent income supporting 3 children, some economic strain |
| Insurance Status (e.g., un/under/insured, public/private, HMO/PPO) | Insured, public |
| Habits (i.e., diet, exercise, caffeine, smoking, alcohol, drugs) | All may be used |
| Activities (i.e., hobbies, sports, clubs, friends) | All may be used |
| Typical day - what is the usual daily routine | All may be used |

| CASE INFORMATION | |
| --- | --- |
| Chief Concern: | Headaches |
| Additional Concerns: | The parent is concerned that headaches are not improving with the treatment recommended at the last visit and is demanding imaging be obtained to rule out “something bad.” |
|  | |
| THE PATIENT STORY: | As the adolescent patient, you are withdrawn and make poor eye contact. You are annoyed by your parent’s concern and just want “to be left alone”. You did not see the point in making changes in your lifestyle that were recommended at the last visit-you do not sleep well, drink pop and coffee daily and do not eat breakfast every day.  As the parent, you are very concerned that headaches are not getting better and something worse could be going on. The relative of one of your close friends died of a brain tumor and the only symptom the person had was headache before the tumor was discovered. |
| HISTORY OF PRESENT ILLNESS:  The patient is a 16-year-old young lady who had been referred six months prior for neurological evaluation as she had been having near daily headaches for three months. The headaches were associated to nausea, emesis, and vision changes. Using over-the-counter medication did not have the desired effect. The headaches lasted several hours, or until the patient slept for two hours. The patient had missed several school days due to headaches. The rest of the patient’s medical history and clinical exam were unremarkable.  At the end of the aforementioned visit, a medication aimed at preventing headaches was prescribed. The medication had the desired effect until two weeks before this visit when the patient began experiencing frequent headaches again.  The conversation should follow one of two paths:  1. If the resident states an MRI is not indicated or an MRI is not brought up in conversation, you will push for the girl to undergo MRI of the brain. The physician explains that considering the nature of the headaches, the normal exam, and the initial success of the treatment strategy, an MRI is not indicated. Furthermore, the physician indicates that assessment is based on the aforementioned and the practice guidelines set forth by the American Academy of Neurology. Your resolve is unchanged, you want an MRI for your daughter. You become progressively upset.  2. If the resident initiates recommendation of an MRI, you should raise concern that they don’t want all those tests – it’s just a headache. You can also take the position of distrust that the doctor is just trying to charge them more for fancy tests, not wanting incidental results, cost, time.  In either scenario be SP should push back 3 times and then acquiesce. | |
|  | |
| REVIEW OF SYSTEMS: Significant positives and negatives | |
| None | |
| Past medical history |  |
| Medication allergies (Name and reaction) | NKDA |
| Environmental allergies (Name and reaction) | none |
| Illnesses | Healthy apart from migraine headaches.  If the resident asks of about a history of anxiety or depression, the teenager should get defensive and say the doctor thinks “it is all in my head” or that “I am making up the headaches” |
| Vaccinations | Up to date |
| Surgeries | None |
| Accidents/ injuries/ trauma | None |
| Hospitalization | None |
|  | |
| Inclusive sexual and reproductive history | |
| Sexual practices  Sexual partners  Protection: Use of safer sex practices  Use of birth control if appropriate  Risk of intimate partner violence | Not sexually active |
| Ob/GYN HISTORY | N/A |
| Medications | over-the-counter NSAIDS as needed for headaches |
| Immunizations | X up to date |
| Tobacco products:   - Cigarettes - Cigar - Pipe - Chew - E-cigarettes | X Never   - Past- year started/year quit - Current   - Quantity   - # of years |
| Alcohol   - Beer - Wine - Liquor - Other | X Never   - Past- year started/year quit - Current   - Quantity   - # of years |
| Drugs   - Weed - Cocaine - Heroin - Meth - Other - IV - Inhalants - Other | X Never   - Past- year started/year quit - Current   - Quantity - # of years |
| Diet (describe) | Typical American diet |
| Exercise (describe) | Not physically active |
| List any other important social history or information important to this case | The patient is currently a sophomore in high school, lives with 2 younger siblings and single parent. Reports that she “worries about her grades”, but is not “stressed” |
| Family history |  |
| Mother, Father, Siblings, Grandparents, and other significant findings. | Mother has migraine headaches, 2 younger siblings are healthy |
|  |  |
| Physical Exam-  *Residents were not asked to complete a neurological exam.* | |
| PHYSICAL EXAM FINDINGS | None |
|  |  |
| DIAGNOSIS AND DIFFERENTIAL | Diagnosis is known to the learners |
|  |  |
| MANAGEMENT OR DIAGNOSTIC PLAN | The patient has common migraine headaches without any red flag symptoms. In addition, she has several lifestyle factors that are likely contributing to the increased frequency of headaches. The next step in her management would be to improve her headache hygiene. Given the absence of red flag symptoms, neuroimaging would not be indicated |
| PROFESSIONALISM ISSUES OR CHALLENGES: | The resident must reassure the patient and her mother and assuage anxiety while not giving in to parent demands for unnecessary testing |

**Veronica Door Instructions**

A 16-year-old young lady and her parent come to clinic for follow-up. She had been referred six months prior for neurological evaluation as she had been having near daily headaches for three months. The headaches were associated to nausea, emesis, and vision changes. At the initial visit she had a normal neurological exam and you diagnosed the patient as having migraines. Using over-the-counter medication did not have the desired effect. The headaches lasted several hours, or until the patient slept for two hours. The patient had missed several school days due to headaches. The rest of the patient’s medical history and clinical exam were unremarkable.

At the end of the last visit, a medication aimed at preventing headaches was prescribed. The medication had the desired effect until two weeks prior when the patient began experiencing frequent headaches again. Now she returns to discuss her plan of care.

Please speak with the patient and her parent regarding next steps in her care as well as answer any questions they may have.

*Please keep in mind that you will have 20 minutes to complete the discussion. Also, please remember that you will be given feedback on how you communicate with the parent, not the content of that discussion or your clinical knowledge.*
