# Supplementary material for: Improving Child Neurology Residents' Communication Skills Through Objective Structured Clinical Exams
Source: MedEdPORTAL. 2021 Mar 4;17:11120. doi: 10.15766/mep_2374-8265.11120 (PMC7970633; doi:10.15766/mep_2374-8265.11120)
Supplement: Supplementary file 1 — Acute Stroke Scenario.docxMedical Error Scenario.docxStaring Spells Scenario.docxTourette Scenario.docxMigraine Scenario.docxDevelopmental Delay Scenario.docxDeath by Neurologic Criteria Scenario.docxPsychogenic Nonepileptic Events Scenario.docxNeonatal Hypoxic Ischemic Encephalopathy Scenario.docxFaculty & SP Assessment Form.docxLearner Self-Assessment Form.docxPost-OSCE Survey.docx [file mep_2374-8265.11120-s001.zip › F. Developmental Delay Scenario.docx]

**Child Neuro OSCE Case 6: Developmental delay (Abbey)**

Date: 12/18/2018

Primary Case Author: Margie Ream

Secondary Case Author: Pedro Weisleder, Dara VF Albert

Standardized Patient Educator: Todd Lash

Name of Case: Developmental delay

Name of educational and or assessment activity: Gap-Kalamazoo Communication Skills Assessment Form, with modifications

Patient Name: Abbey

Chief Complaint: Developmental delay

Most likely Diagnosis and Differential with rationale from history and/or physical exam:

Challenge question:

Domains: Check all that apply

X Professionalism

X Communication and Interpersonal skills

- Medical History
- Physical exam
- Shared Decision Making

X Patient Education

- Clinical Reasoning
- Documentation
- Handoff
- Presentation
- Other:

Type and level of learner: pediatric and adult neurology residents (post-graduate years 2-5)

Case Objectives: please list specific objectives for each of the domains you have checked above:

1. Reassure family that everything has been done to evaluate the cause of the problems, but that sometimes an answer cannot be found.

2. Demonstrate communication skills when there is not a specific diagnosis.

3. Help the family accept uncertainty.

| SETTING: | outpatient Neurology Clinic |
| --- | --- |
| PATIENT PROFILE: | |
| Age range | The patient is 5 years old, parents are in late-30s (this case can use a single parent or a two-parent dyad) |
| Religious/spiritual background | All may be used |
| Sex (e.g., male, female, intersex, transwoman, transman) | All may be used |
| Sexual Orientation (e.g., heterosexual, lesbian, gay, bisexual, pansexual, queer, asexual) | All may be used |
| Gender expression (e.g., man, woman, gender queer) | All may be used |
| Race/ethnicity: | All may be used |
| Physical description (e.g., BMI, height range) | All may be used |
| Physical limitations | All may be used |
| Patient appearance (e.g., disheveled, hospital gown, business casual, casual) | All may be used |
| Moulage + location (e.g., none, bruises, scars, body piercing, tattoos) | None |
| Affect (e.g., pleasant, cooperative) | Initially parent(s) are anxious to hear the results of the test they were expecting would provide the final diagnosis, and are disappointed to learn that no specific diagnosis has been made |
| Family group (e.g., who is family, who they live with) | All may be used |
| Education | Both parents are college-educated |
| Level of health literacy | Moderate |
| Employment, if any - present and past, noting any current stresses | Both parents are dedicated to their careers and wanted to wait until they were “ready” to have children. While they have good jobs they are middle class and struggling to afford the bills from the medical expenses and potential for long term care for their child |
| Home/homeless - type of dwelling, number of stories, owned or rented | All may be used |
| Financial situation- any current stresses | All may be used |
| Insurance Status (e.g., un/under/insured, public/private, HMO/PPO) | All may be used |
| Habits (i.e., diet, exercise, caffeine, smoking, alcohol, drugs) | All may be used |
| Activities (i.e., hobbies, sports, clubs, friends) | All may be used |
| Typical day - what is the usual daily routine | All may be used |

| CASE INFORMATION | |
| --- | --- |
| Chief Concern: | Developmental delay |
| Additional Concerns: | The child is significantly delayed and at 5 years of age cannot walk, stand or crawl. The child grunts and points but does not speak. |
|  | |
| THE PATIENT STORY: | As the parents, you are very distraught that you have spent nearly 5 years and thousands of dollars and don’t have any answer. You delayed child bearing until you were ready and now you feel that their hopes are dashed and are worried about the risk of recurrence if they were to try for another child. You also want to know how to help the child but since there is no diagnosis feel like the doctors don’t know anything. How can doctors at such a major pediatric hospital not know what is wrong?!?  In your frustration, you have considered traveling the country seeking additional opinions from other Children's hospitals. |
| HISTORY OF PRESENT ILLNESS:  The child was born after an ideal pregnancy; there were no concerns until 2 months of age when pediatrician noted child was not improving in head control. There were some difficulties with feeding too that mom blamed on her own milk production so gave up on breast feeding and switched to bottle early in the newborn period. By 6 months it was obvious the child was severely delayed – still no improvement in head control, no cooing, minimal interaction with environment but eating and gaining weight well. Now at 5 years old the child has achieved the developmental level of a 6-9 month old. S/he can sit but cannot walk. She babbles some but doesn’t have any specific words. She gets her nutrition from a sippy cup and baby foods.  Parents began the diagnostic odyssey shortly after 6 months well child check and has seen specialists in genetics, developmental pediatrics and neurology. Testing has included extensive metabolic testing, MRI, whole exome sequence, mitochondrial genome sequencing and research based whole genome sequencing. They return to clinic today for results of the WGS. All tests were normal. There is no answer as to why the child has the disabilities she has. | |
|  | |
| REVIEW OF SYSTEMS: Significant positives and negatives | |
| Negative | |
| Past medical history |  |
| Medication allergies (Name and reaction) | NKDA |
| Environmental allergies (Name and reaction) | None |
| Illnesses | Developmental delay |
| Vaccinations | Up to date |
| Surgeries | None |
| Accidents/ injuries/ trauma | None |
| Hospitalization | None |
|  | |
| Inclusive sexual and reproductive history | |
| Sexual practices  Sexual partners  Protection: Use of safer sex practices  Use of birth control if appropriate  Risk of intimate partner violence | N/A |
| Ob/GYN HISTORY | N/A |
| Medications | None |
| Immunizations | X up to date |
| Tobacco products:   - Cigarettes - Cigar - Pipe - Chew - E-cigarettes | X Never   - Past- year started/year quit - Current   - Quantity   - # of years |
| Alcohol   - Beer - Wine - Liquor - Other | Mom had 1 glass of wine per week until she realized she was pregnancy at 6 weeks and wonders if that lead to the delays in her daughter  X Never   - Past- year started/year quit - Current   - Quantity   - # of years |
| Drugs   - Weed - Cocaine - Heroin - Meth - Other - IV - Inhalants - Other | X Never   - Past- year started/year quit - Current   - Quantity - # of years |
| Diet (describe) | Child drinks pediasure from a sippy cup, some spoon fulls of baby food |
| Exercise (describe) | Active in therapies |
| List any other important social history or information important to this case | Both parents are dedicated to their careers and wanted to wait until they were “ready” to have children. While they have good jobs they are middle class and struggling to afford the bills from the medical expenses and potential for long term care for their child. |
| Family history |  |
| Mother, Father, Siblings, Grandparents, and other significant findings. | Father has well-controlled diabetes  The patient has no siblings  Maternal grandmother has Parkinson's disease |
|  |  |
| Physical Exam-  *Residents were not asked to complete a neurological exam.* | |
| PHYSICAL EXAM FINDINGS | None |
|  |  |
| DIAGNOSIS AND DIFFERENTIAL | Diagnosis is not known to the learners |
|  |  |
| MANAGEMENT OR DIAGNOSTIC PLAN | Abbey has significant global developmental delay of unknown etiology. |
|  |  |
| PROFESSIONALISM ISSUES OR CHALLENGES: | At this point, the medical team has exhausted all potential diagnostic investigations and the resident needs to help the family accept the lack of diagnosis. |

**Abbey Door Instructions**

Abbey is a 5-year-old child with developmental delay who has had a very thorough diagnostic evaluation, which has not revealed the cause of the delays. The family is frustrated at having supposedly the best medical care but still having no answers. They are wanting more testing – surely there must be an answer as to why their child is not normal. Testing has included extensive metabolic testing, MRI, whole exome sequence, mitochondrial genome sequencing and research based whole genome sequencing. They return to clinic today for results of the WGS. All tests were normal. There is no answer as to why the child has the disabilities she has. You have exhausted all testing options that are available at this point in medical science and the family have to accept that their child is disabled without knowing why.

Explain to the family and reassure them that everything has been done to evaluate the cause of the problems but that sometimes an answer cannot be found.

*Please keep in mind that you will have 20 minutes to complete the discussion. Also, please remember that you will be given feedback on how you communicate with the parents, not the content of that discussion or your clinical knowledge.*
