# Supplementary material for: Improving Child Neurology Residents' Communication Skills Through Objective Structured Clinical Exams
Source: MedEdPORTAL. 2021 Mar 4;17:11120. doi: 10.15766/mep_2374-8265.11120 (PMC7970633; doi:10.15766/mep_2374-8265.11120)
Supplement: Supplementary file 1 — Acute Stroke Scenario.docxMedical Error Scenario.docxStaring Spells Scenario.docxTourette Scenario.docxMigraine Scenario.docxDevelopmental Delay Scenario.docxDeath by Neurologic Criteria Scenario.docxPsychogenic Nonepileptic Events Scenario.docxNeonatal Hypoxic Ischemic Encephalopathy Scenario.docxFaculty & SP Assessment Form.docxLearner Self-Assessment Form.docxPost-OSCE Survey.docx [file mep_2374-8265.11120-s001.zip › G. Death by Neurologic Criteria Scenario.docx]

**Child Neuro OSCE Case 7: Death by Neurologic Criteria (Brook)**

Date: 12/17/2018

Primary Case Author: Pedro Weisleder

Secondary Case Author: Margie Ream, Dara VF Albert

Standardized Patient Educator: Todd Lash

Name of Case: Death by Neurologic Criteria

Name of educational and or assessment activity: Gap-Kalamazoo Communication Skills Assessment Form, with modifications

Patient Name: Brook

Chief Complaint:

Most likely Diagnosis and Differential with rationale from history and/or physical exam:

Challenge question:

Domains: Check all that apply

X Professionalism

X Communication and Interpersonal skills

- Medical History
- Physical exam
- Shared Decision Making

X Patient Education

- Clinical Reasoning
- Documentation
- Handoff
- Presentation
- Other:

Type and level of learner: pediatric and adult neurology residents (post-graduate years 2-5)

Case Objectives: please list specific objectives for each of the domains you have checked above:

1. The resident needs to explain to the parents the concept of death by neurological criteria, and how physicians make that determination.

2. Demonstrate the skills necessary to deliver such terrible news.

3. Demonstrate empathy and compassion in the delivery of grim news.

| SETTING: | pediatric intensive care unit |
| --- | --- |
| PATIENT PROFILE: | |
| Age range | Patient is 2 years old. Parents are in their early 30s |
| Religious/spiritual background | All may be used |
| Sex (e.g., male, female, intersex, transwoman, transman) | All may be used |
| Sexual Orientation (e.g., heterosexual, lesbian, gay, bisexual, pansexual, queer, asexual) | All may be used |
| Gender expression (e.g., man, woman, gender queer) | All may be used |
| Race/ethnicity: | All may be used |
| Physical description (e.g., BMI, height range) | All may be used |
| Physical limitations | All may be used |
| Patient appearance (e.g., disheveled, hospital gown, business casual, casual) | All may be used |
| Moulage + location (e.g., none, bruises, scars, body piercing, tattoos) | None |
| Affect (e.g., pleasant, cooperative) | Parents are guilt-ridden. They know that an adult should have been paying attention to Brook. However, father was cooking food on the grill, and mother was working on the finishing touches of the birthday cake.  Parents are quiet. Every time they try to speak, they start crying. The mood in the room is somber. |
| Family group (e.g., who is family, who they live with) | All may be used |
| Education | A couple years of college education but did not graduate from college. |
| Level of health literacy | Moderate |
| Employment, if any - present and past, noting any current stresses | Father has a midlevel managerial job. Mother has a part-time clerical job. |
| Home/homeless - type of dwelling, number of stories, owned or rented | All may be used |
| Financial situation- any current stresses | All may be used |
| Insurance Status (e.g., un/under/insured, public/private, HMO/PPO) | All may be used |
| Habits (i.e., diet, exercise, caffeine, smoking, alcohol, drugs) | All may be used |
| Activities (i.e., hobbies, sports, clubs, friends) | All may be used |
| Typical day - what is the usual daily routine | All may be used |

| CASE INFORMATION | |
| --- | --- |
| Chief Concern: | Near-drowning |
| Additional Concerns: |  |
|  | |
| THE PATIENT STORY: | You are the parents of a 2-year-old girl that was found face down in your neighbor’s swimming pool. You are incredibly grief-stricken as well as guilty that the child managed to escape your supervision. You understand that her situation is dire, but still hope for a miracle. You hang on every glimmer of positive news and hyper-fixate on little details so you do not have to grasp the “big picture”. You also worry about how you will explain this to your 5-year-old.  You don’t understand why is it that the doctors are telling you that Brook is “dead,” when her heart is beating, and the ventilator is breathing for her. Also, the doctors keep telling you that all blood work is normal.  You don’t understand how the diagnosis of “brain death” can be made without doing additional testing.  You should push for additional testing 3 times, but eventually accept that a physical exam is needed, and that the results of the exam are final. |
| HISTORY OF PRESENT ILLNESS:  Brook is a 2-year-old girl with no significant past medical history who was brought to the hospital after she was found face down in a pool. The child had been at her brother’s birthday party in the backyard of her house. Brook’s parents did not realize that the girl had wondered off to the neighbors’ backyard, and that she had fallen into their pool. The family estimates that the child was under no adult supervision for 15-20 minutes. The paramedics tried to resuscitate Brook in the field. Only after 30 minutes of intensive resuscitation, were they able to get a pulse.  The girl’s condition has deteriorated over the past 48 hours. At the time of admission to the PICU, the girl’s pupils were responding to light, albeit slowly. The girl also had corneal reflexes, and a weak cough reflex. Alas, those reflexes have disappeared. An MRI of the brain completed on the morning after the admission, reveled severe and diffuse hypoxic-ischemic injury, and impending signs of central herniation. Twelve hours ago, the PICU team completed the first evaluation to determine function of the brain and brainstem. The test made clear that the patient had findings compatible with death by neurological criteria. You were asked to complete the second confirmatory exam. Before doing so, you want to speak with Brook’s parents and explain to them the notion of “brain death,” and its medical and legal implications. | |
|  | |
| REVIEW OF SYSTEMS: Significant positives and negatives | |
| Respiratory failure, hypotensive, unresponsive | |
| Past medical history |  |
| Medication allergies (Name and reaction) | NKDA |
| Environmental allergies (Name and reaction) | None |
| Illnesses | None previously |
| Vaccinations | Up to date |
| Surgeries | None |
| Accidents/ injuries/ trauma | None |
| Hospitalization | None prior to current |
|  | |
| Inclusive sexual and reproductive history | |
| Sexual practices  Sexual partners  Protection: Use of safer sex practices  Use of birth control if appropriate  Risk of intimate partner violence | N/A |
| Ob/GYN HISTORY | N/A |
| Medications | None |
| Immunizations | X up to date |
| Tobacco products:   - Cigarettes - Cigar - Pipe - Chew - E-cigarettes | X Never   - Past- year started/year quit - Current   - Quantity   - # of years |
| Alcohol   - Beer - Wine - Liquor - Other | X Never   - Past- year started/year quit - Current   - Quantity   - # of years |
| Drugs   - Weed - Cocaine - Heroin - Meth - Other - IV - Inhalants - Other | X Never   - Past- year started/year quit - Current   - Quantity - # of years |
| Diet (describe) | Typical American diet |
| Exercise (describe) | Normal toddler activity |
| List any other important social history or information important to this case | The drowning occurred during the 5-year-old brother’s birthday party |
| Family history |  |
| Mother, Father, Siblings, Grandparents, and other significant findings. | Parents and 5-year-old brother are healthy |
|  |  |
| Physical Exam-  *Residents were not asked to complete a neurological exam.* | |
| PHYSICAL EXAM FINDINGS | None |
|  |  |
| DIAGNOSIS AND DIFFERENTIAL | Diagnosis is known to the learners |
|  |  |
| MANAGEMENT OR DIAGNOSTIC PLAN | The child has suffered severe hypoxic ischemic injury to the brain and is likely meeting neurologic criteria for brain death based on the initial “brain death testing”. She will need to undergo the 2nd, confirmatory test. |
|  |  |
| PROFESSIONALISM ISSUES OR CHALLENGES: | The resident will need to explain to the parents the concept of death by neurological criteria and how physicians make that determination. |

**Brook Door Instructions**

Brook is a 2-year-old girl with no significant past medical history who was brought to the hospital after she was found face down in a pool. The child had been at her brother’s birthday party in the backyard of her house. Brook’s parents did not realize that the girl had wondered off to the neighbors’ backyard, and that she had fallen into their pool. The family estimates that the child was under no adult supervision for 15-20 minutes. The paramedics tried to resuscitate Brook in the field. Only after 30 minutes of intensive resuscitation, were they able to get a pulse.

The girl’s condition has deteriorated over the past 48 hours. At the time of admission to the PICU, the girl’s pupils were responding to light, albeit slowly. The girl also had corneal reflexes, and a weak cough reflex. Alas, those reflexes have disappeared. An MRI of the brain completed on the morning after the admission, reveled severe and diffuse hypoxic-ischemic injury, and impending signs of central herniation. Twelve hours ago, the PICU team completed the first evaluation to determine function of the brain and brainstem. The test made clear that the patient had findings compatible with death by neurological criteria. You were asked to complete the second confirmatory exam. Before doing so, you want to speak with Brook’s parents and explain to them the notion of “brain death,” and its medical and legal implications.

Explain to the parents that the child is suspected to be “brain dead.” The next step is to complete the second formal test necessary to confirm death by neurological criteria. If as presumed, the child has no brain function, she will be legally dead, and all medically provided treatments will need to stop. Explain to the parents the concept of death by neurological criteria, and how physicians make that determination.

*Please keep in mind that you will have 20 minutes to complete the discussion. Also, please remember that you will be given feedback on how you communicate with the parents, not the content of that discussion or your clinical knowledge.*
