# Supplementary material for: Improving Child Neurology Residents' Communication Skills Through Objective Structured Clinical Exams
Source: MedEdPORTAL. 2021 Mar 4;17:11120. doi: 10.15766/mep_2374-8265.11120 (PMC7970633; doi:10.15766/mep_2374-8265.11120)
Supplement: Supplementary file 1 — Acute Stroke Scenario.docxMedical Error Scenario.docxStaring Spells Scenario.docxTourette Scenario.docxMigraine Scenario.docxDevelopmental Delay Scenario.docxDeath by Neurologic Criteria Scenario.docxPsychogenic Nonepileptic Events Scenario.docxNeonatal Hypoxic Ischemic Encephalopathy Scenario.docxFaculty & SP Assessment Form.docxLearner Self-Assessment Form.docxPost-OSCE Survey.docx [file mep_2374-8265.11120-s001.zip › H. Psychogenic Nonepileptic Events Scenario.docx]

**Child Neuro OSCE Case 8: Psychogenic Nonepileptic Events (Bradley)**

Date: 11/9/2018

Primary Case Author: Dara VF Albert

Secondary Case Author: Margie Ream, Pedro Weisleder

Standardized Patient Educator: Todd Lash

Name of Case: Psychogenic Nonepileptic Events

Name of educational and or assessment activity: Gap-Kalamazoo Communication Skills Assessment Form, with modifications

Patient Name: Bradley

Chief Complaint: seizure-like episodes

Most likely Diagnosis and Differential with rationale from history and/or physical exam: Psychogenic Nonepileptic Events

Challenge question: The resident needs to convey the diagnosis to the adolescent and his parent.

Domains: Check all that apply

X Professionalism

X Communication and Interpersonal skills

- Medical History
- Physical exam
- Shared Decision Making

X Patient Education

- Clinical Reasoning
- Documentation
- Handoff
- Presentation
- Other:

Type and level of learner: pediatric and adult neurology residents (post-graduate years 2-5)

Case Objectives: please list specific objectives for each of the domains you have checked above:

1. Provide reassurance to patient and parent(s) as well as gain acceptance of the diagnosis.

2. Demonstrate communication skills in what could be an adversarial situation with a challenging diagnosis.

3. Demonstrate empathy and seek to understand the parent/family prospective regarding treatment

| SETTING: | Inpatient neurology ward |
| --- | --- |
| PATIENT PROFILE:  (the case was written for a male adolescent patient; however pronouns could be interchangeable and the name could be changed if a female patient is preferred) | |
| Age range | The patient is a 16-year-old, the parent is in late-40s (the case is written as the patient is a male with his mother, but can also be a female patient and/or a male parent) |
| Religious/spiritual background | All may be used |
| Sex (e.g., male, female, intersex, transwoman, transman) | All may be used |
| Sexual Orientation (e.g., heterosexual, lesbian, gay, bisexual, pansexual, queer, asexual) | All may be used |
| Gender expression (e.g., man, woman, gender queer) | All may be used |
| Race/ethnicity: | All may be used |
| Physical description (e.g., BMI, height range) | All may be used |
| Physical limitations | All may be used |
| Patient appearance (e.g., disheveled, hospital gown, business casual, casual) | All may be used |
| Moulage + location (e.g., none, bruises, scars, body piercing, tattoos) | None |
| Affect (e.g., pleasant, cooperative) | The parent is irritable as the previous doctor told them the adolescent’s symptoms were “all in his/her head.” The patient is initially flat but feeds off parent’s anger/anxiety and becomes angrier. |
| Family group (e.g., who is family, who they live with) | All may be used |
| Education | The patient is currently in high school, the parent graduated high school |
| Level of health literacy | Low to moderate |
| Employment, if any - present and past, noting any current stresses | The patient is in high school, the parent has a clerical job |
| Home/homeless - type of dwelling, number of stories, owned or rented | All may be used |
| Financial situation- any current stresses | Single-parent household with 3 kids, low-paying job |
| Insurance Status (e.g., un/under/insured, public/private, HMO/PPO) | Insured, public |
| Habits (i.e., diet, exercise, caffeine, smoking, alcohol, drugs) | All may be used |
| Activities (i.e., hobbies, sports, clubs, friends) | All may be used |
| Typical day - what is the usual daily routine | All may be used |

| CASE INFORMATION | |
| --- | --- |
| Chief Concern: | Seizure-like episodes |
| Additional Concerns: |  |
|  | |
| THE PATIENT STORY: | The parent is very concerned about these events, especially after watching his/her father suffer from epilepsy his whole life. The patient was seen at an outside facility and was told the events were “all in patient’s head.” You are very angry and are certain there must be something else going on, therefore are seeking another opinion.  If asked about anxiety or depression, as the teenager, you should get defensive and say the doctor thinks “it’s all in my head,” but should listen if the resident explains the relationship between these events and anxiety/depression. |
| HISTORY OF PRESENT ILLNESS:  The patient is admitted to the inpatient neurology service for seizure-like events. The adolescent has events consisting of whole body shaking and unresponsiveness lasting 30-60 minutes typically occurring in the middle of the school day. The events look different every time and there is no warning prior to their onset. The events can wax and wane in intensity and character (i.e. one event might consist of “flopping around like a fish” while others s/he can be totally still and unresponsive). Patient was seen at an outside hospital and was told “these events are all in your head” and to “just ignore them.” They did not do any tests, just sent patient home. The parent and patient are angry.  In this scenario, the SP should push back on accepting the diagnosis 3 times and then acquiesce. Once parent accepts the diagnosis, should ask what do we do at home or at school when the events continue? Patient should ask how do I control the events? | |
|  | |
| REVIEW OF SYSTEMS: Significant positives and negatives | |
| Intermittent headaches | |
| Past medical history |  |
| Medication allergies (Name and reaction) | NKDA |
| Environmental allergies (Name and reaction) | None |
| Illnesses | Seizure-like events |
| Vaccinations | Up to date |
| Surgeries | None |
| Accidents/ injuries/ trauma | None |
| Hospitalization | None |
|  | |
| Inclusive sexual and reproductive history | |
| Sexual practices  Sexual partners  Protection: Use of safer sex practices  Use of birth control if appropriate  Risk of intimate partner violence | No (if asked without mom in the room, will admit to having sex with significant other for the first time right before they broke up, used a condom) |
| Ob/GYN HISTORY | N/A |
| Medications |  |
| Immunizations | X up to date |
| Tobacco products:   - Cigarettes - Cigar - Pipe - Chew - E-cigarettes | X Never   - Past- year started/year quit - Current   - Quantity   - # of years |
| Alcohol   - Beer - Wine - Liquor - Other | X Never   - Past- year started/year quit - Current   - Quantity   - # of years |
| Drugs   - Weed - Cocaine - Heroin - Meth - Other - IV - Inhalants - Other | X Never   - Past- year started/year quit - Current   - Quantity - # of years |
| Diet (describe) | Typical American diet |
| Exercise (describe) | Active in sports |
| List any other important social history or information important to this case | Parent is a single parent. The patient is single, recently broke up with his/her significant other. The Adolescent is a straight A student who “puts a lot of pressure on him/herself.” He/she is involved in football, soccer, piano and volunteers in the community with the family’s church.  Patient is “over-scheduled” with many school and extra-curricular pressures.  Maternal grandfather recently past, he had seizures his whole life |
| Family history |  |
| Mother, Father, Siblings, Grandparents, and other significant findings. | Mother has anxiety, father no longer in their lives and details of his or his relatives’ history are not known  Patient has 2 healthy siblings  Maternal grandfather recently passed due to a heart attack, he had epilepsy since childhood  Maternal grandmother is alive and has well-controlled diabetes |
|  |  |
| Physical Exam-  *Residents were not asked to complete a neurological exam.* | |
| PHYSICAL EXAM FINDINGS | None |
|  |  |
| DIAGNOSIS AND DIFFERENTIAL | Diagnosis is known to the learners |
|  |  |
| MANAGEMENT OR DIAGNOSTIC PLAN | The patient has psychogenic nonepileptic events that have been confirmed on EEG. |
|  |  |
| PROFESSIONALISM ISSUES OR CHALLENGES: | The next step is to share the diagnosis with the patient and parent and help them accept the diagnosis in order to stop the diagnostic odyssey and move towards treatment. |

**Bradley Door Instructions**

Bradley is a 16-year-old adolescent admitted last night to the neurology service for evaluation of seizure-like events that started about 3 months ago. He was seen at an outside facility and was told the events were “all in patient’s head.” The parent is very angry and is certain there must be something else going on, therefore is seeking another opinion from the experts at your hospital.

Overnight Bradley was connected to a long-term video EEG and several typical events were captured. These events were not stereotyped and had no abnormal electrographic correlate, suggesting they are psychogenic non-epileptic events.

Explain to the family the diagnosis and provide reassurances to facilitate acceptance of the diagnosis.

*Please keep in mind that you will have 20 minutes to complete the discussion. Also, please remember that you will be given feedback on how you communicate with the parents, not the content of that discussion or your clinical knowledge.*
