# Supplementary material for: Improving Child Neurology Residents' Communication Skills Through Objective Structured Clinical Exams
Source: MedEdPORTAL. 2021 Mar 4;17:11120. doi: 10.15766/mep_2374-8265.11120 (PMC7970633; doi:10.15766/mep_2374-8265.11120)
Supplement: Supplementary file 1 — Acute Stroke Scenario.docxMedical Error Scenario.docxStaring Spells Scenario.docxTourette Scenario.docxMigraine Scenario.docxDevelopmental Delay Scenario.docxDeath by Neurologic Criteria Scenario.docxPsychogenic Nonepileptic Events Scenario.docxNeonatal Hypoxic Ischemic Encephalopathy Scenario.docxFaculty & SP Assessment Form.docxLearner Self-Assessment Form.docxPost-OSCE Survey.docx [file mep_2374-8265.11120-s001.zip › I. Neonatal Hypoxic Ischemic Encephalopathy Scenario.docx]

**Child Neuro OSCE Case 9: Neonatal Hypoxic-Ischemic Encephalopathy (Finn)**

Date: 3/1/2018

Primary Case Author: Margie Ream

Secondary Case Author: Dara VF Albert, Pedro Weisleder

Standardized Patient Educator: Todd Lash

Name of Case: Neonatal Hypoxic-Ischemic Encephalopathy

Name of educational and or assessment activity: Gap-Kalamazoo Communication Skills Assessment Form, with modifications

Patient Name: “Finn” Baby Boy Brown

Chief Complaint: neonatal distress

Most likely Diagnosis and Differential with rationale from history and/or physical exam: Baby has suffered severe hypoxic-ischemic injury during delivery/after birth.

Challenge question:

Domains: Check all that apply

X Professionalism

X Communication and Interpersonal skills

- Medical History
- Physical exam
- Shared Decision Making

X Patient Education

- Clinical Reasoning
- Documentation
- Handoff
- Presentation
- Other:

Type and level of learner: pediatric and adult neurology residents (post-graduate years 2-5)

Case Objectives:

1. Discuss goals of care

2. Demonstrate communication skills and deliver bad news regarding prognosis.

3. Demonstrate empathy and compassion while delivering bad news regarding grim prognosis

| SETTING: | neonatal intensive care unit |
| --- | --- |
| PATIENT PROFILE: | |
| Age range | The baby is a neonate, parents are in their 30s |
| Religious/spiritual background | All may be used |
| Sex (e.g., male, female, intersex, transwoman, transman) | All may be used |
| Sexual Orientation (e.g., heterosexual, lesbian, gay, bisexual, pansexual, queer, asexual) | All may be used |
| Gender expression (e.g., man, woman, gender queer) | All may be used |
| Race/ethnicity: | All may be used |
| Physical description (e.g., BMI, height range) | All may be used |
| Physical limitations | All may be used |
| Patient appearance (e.g., disheveled, hospital gown, business casual, casual) | All may be used |
| Moulage + location (e.g., none, bruises, scars, body piercing, tattoos) | None |
| Affect (e.g., pleasant, cooperative) | Mom is very sad, dad is anxious and somewhat combative |
| Family group (e.g., who is family, who they live with) | All may be used |
| Education | Both parents are highly educated (graduate degrees)  Mom is a consultant for small businesses, dad is an engineer |
| Level of health literacy | Parents have no experience with pediatric medical care or critical care |
| Employment, if any - present and past, noting any current stresses | Highly educated dual career parents |
| Home/homeless - type of dwelling, number of stories, owned or rented | All may be used |
| Financial situation- any current stresses | All may be used |
| Insurance Status (e.g., un/under/insured, public/private, HMO/PPO) | All may be used |
| Habits (i.e., diet, exercise, caffeine, smoking, alcohol, drugs) | All may be used |
| Activities (i.e., hobbies, sports, clubs, friends) | All may be used |
| Typical day - what is the usual daily routine | All may be used |

| CASE INFORMATION | |
| --- | --- |
| Chief Concern: | Newborn baby is not waking up or breathing on his own |
| Additional Concerns: | Full term delivery with prolonged vacuum assistance. Baby was "stuck" for a few minutes.  Baby looks perfect but is not reactive except for occasional brief foot movement that is likely a reflex. Baby is intubated |
|  | |
| THE PATIENT STORY: | You are both highly (non-medical) educated 1st-time parents. You can tell that your baby is very sick, but do not fully understand the big picture. The resident comes to talk to you about your baby. You ask if baby is brain dead. Until this conversation you did not understand that baby had such severe brain injury.  You initially start with questions about high level functions, like will he be able to read in 1st grade or play on the soccer team. As the conversation goes on you lower your expectations and wonder if the baby will every walk or talk, then if the baby will breathe or eat by mouth.  As the mom, you express guilt over not being able to prevent this, and guilt that you feel like it is your fault because you had a glass of champagne the night before delivery to celebrate a major accomplishment at work.  As the dad you fixate on irrelevant details like how often the pulse oximetry readings might be unreliable because the probe falls off and if the hypothermia machine was set to a low enough temperature to protect baby’s brain. This is your “defense mechanism” as a way of not breaking down entirely. You should focus on these little details at least 3 times in the conversation, then allow yourself to be sad. |
| HISTORY OF PRESENT ILLNESS:  A full-term baby is born to first time parents. The baby has hypoxic ischemic encephalopathy due to unexpected delivery complications (there is a knot in the umbilical cord). The baby needs extensive resuscitation at birth and is intubated in the first few minutes of life. After 3 days of therapeutic hypothermia and rewarming there is minimal brain activity on EEG (burst suppression pattern), MRI is consistent with diffuse severe injury and baby is starting to breathe on his own but not well enough to survive off the ventilator.  Resident picks up care where the previous day’s team left off. The parents are aware of poor brain activity and the cause of the injury. The goal of the encounter is for the resident to make sure the parents understand that the likelihood of meaningful brain recovery is very low and that the family will need to decide if they will allow natural death and extubate or if they will pursue G-tube and possibly tracheostomy. | |
|  | |
| REVIEW OF SYSTEMS: Significant positives and negatives | |
| Intubated, ventilated, not currently being fed | |
| Past medical history |  |
| Medication allergies (Name and reaction) | NKDA |
| Environmental allergies (Name and reaction) | None |
| Illnesses | None |
| Vaccinations | Up to date |
| Surgeries | None |
| Accidents/ injuries/ trauma | None |
| Hospitalization | None |
|  | |
| Inclusive sexual and reproductive history | |
| Sexual practices  Sexual partners  Protection: Use of safer sex practices  Use of birth control if appropriate  Risk of intimate partner violence | N/A |
| Ob/GYN HISTORY | N/A |
| Medications | No sedating medications |
| Immunizations | X up to date |
| Tobacco products:   - Cigarettes - Cigar - Pipe - Chew - E-cigarettes | X Never   - Past- year started/year quit - Current   - Quantity   - # of years |
| Alcohol   - Beer - Wine - Liquor - Other | the mother had a glass of champagne the night prior to delivery  X Never   - Past- year started/year quit - Current   - Quantity   - # of years |
| Drugs   - Weed - Cocaine - Heroin - Meth - Other - IV - Inhalants - Other | X Never   - Past- year started/year quit - Current   - Quantity - # of years |
| Diet (describe) | Not currently being fed |
| Exercise (describe) | N/A |
| List any other important social history or information important to this case | Were waiting until the “right time” to have a baby they could really take care of. |
| Family history |  |
| Mother, Father, Siblings, Grandparents, and other significant findings. | Ppaternal grandfather died suddenly of a stroke at 50 years old. Otherwise healthy families. Both parents have nieces and nephews and wanted to have happy healthy families like their sibs have. |
|  |  |
| Physical Exam-  *Residents were not asked to complete a neurological exam.* | |
| PHYSICAL EXAM FINDINGS | None |
|  |  |
| DIAGNOSIS AND DIFFERENTIAL | Diagnosis is known to the learners |
|  |  |
| MANAGEMENT OR DIAGNOSTIC PLAN | The resident should share the dire prognosis with the family and discuss goals of care |
|  |  |
| PROFESSIONALISM ISSUES OR CHALLENGES: | Demonstrate communication skills and deliver bad news regarding a poor prognosis |

**“Finn” Baby Boy Brown Door Instructions**

A full-term baby is born to first time parents. The baby has hypoxic ischemic encephalopathy due to unexpected delivery complications (there is a knot in the umbilical cord). The baby needs extensive resuscitation at birth and is intubated in the first few minutes of life. After 3 days of therapeutic hypothermia and rewarming there is minimal brain activity on EEG (burst suppression pattern), MRI is consistent with diffuse severe injury and baby is starting to breathe on his own but not well enough to survive off the ventilator. The baby has severe neurological injury and a poor prognosis but does not fulfill criteria for death by neurologic criteria.

Please speak with the baby’s parents regarding next steps in his care as well as answer any questions they may have about prognosis. You do not have to discuss making any decisions about care in this conversation (such as withdrawal of life support).

*Please keep in mind that you will have 20 minutes to complete the discussion. Also, please remember that you will be given feedback on how you communicate with the parent, not the content of that discussion or your clinical knowledge.*
