# Supplementary material for: Improving Child Neurology Residents' Communication Skills Through Objective Structured Clinical Exams
Source: MedEdPORTAL. 2021 Mar 4;17:11120. doi: 10.15766/mep_2374-8265.11120 (PMC7970633; doi:10.15766/mep_2374-8265.11120)
Supplement: Supplementary file 1 — Acute Stroke Scenario.docxMedical Error Scenario.docxStaring Spells Scenario.docxTourette Scenario.docxMigraine Scenario.docxDevelopmental Delay Scenario.docxDeath by Neurologic Criteria Scenario.docxPsychogenic Nonepileptic Events Scenario.docxNeonatal Hypoxic Ischemic Encephalopathy Scenario.docxFaculty & SP Assessment Form.docxLearner Self-Assessment Form.docxPost-OSCE Survey.docx [file mep_2374-8265.11120-s001.zip › J. Faculty & SP Assessment Form.docx]

**Faculty and Standardized Patient Assessment Form ^1^***

**How well did the participant do the following?**

**1. Build a relationship (includes the following):**

**1. Greets and shows interest in patient and patient's family**

**2. Uses words that show care and concern throughout the interview**

**3. Uses tone, pace, eye contact, and posture that show care and concern**

**4. Response explicitly to patient and family statements about ideas and feelings**

- Did not use these behaviors
- Used 1 or 2 behaviors
- Used many behaviors, yet missed 1 or more opportunities
- Use these and other behaviors to consistently demonstrate this skill throughout

**2. Opens the discussion) includes the following):**

**1. Allows patient and family to complete opening statement without interruption**

**2. Asks “is there anything else?” to elicit full set of concerns**

**3. Explains and/or negotiates and agenda for the visit**

- Did not use these behaviors
- Used 1 or 2 behaviors
- Used many behaviors, yet missed 1 or more opportunities
- Use these and other behaviors to consistently demonstrate this skill throughout

**3. Get others information (includes the following):**

**1. Begins with patient and family story using open-ended questions (example “tell me about…”)**

**2. Clarifies details as necessary with more specific or “yes/no” questions**

**3. Summarizes and gives family opportunity to correct or add information**

**4. Transitions effectively to additional questions**

- Did not use these behaviors
- Used 1 or 2 behaviors
- Used many behaviors, yet missed 1 or more opportunities
- Use these and other behaviors to consistently demonstrate this skill throughout

**4. Understands the patient's and family's perspective (includes the following):**

**1. Asks about life events, circumstances, other people that might affect health**

**2. Elicits patient and family believes, concerns, and expectations about illness and treatment**

- Did not use these behaviors
- Used 1 or 2 behaviors
- Used many behaviors, yet missed 1 or more opportunities
- Use these and other behaviors to consistently demonstrate this skill throughout

**5. Shares information (includes the following):**

**1. Assesses patient and family understanding of problem and desire for more information**

**2. Explains using words that family can understand**

**3. Asks if family has any questions**

- Did not use these behaviors
- Used 1 or 2 behaviors
- Used many behaviors, yet missed 1 or more opportunities
- Use these and other behaviors to consistently demonstrate this skill throughout

**6. Reaches agreement (includes the following):**

**1. Includes family in choices and decisions to the extent they desire**

**2. Checks for mutual understanding of diagnostic and/or treatment plans**

**3. Asks about acceptability of diagnostic and/or treatment plans**

**4. Identifies additional resources as appropriate**

- Did not use these behaviors
- Used 1 or 2 behaviors
- Used many behaviors, yet missed 1 or more opportunities
- Use these and other behaviors to consistently demonstrate this skill throughout

**7. Provides closure (includes the following):**

**1. Asks if patient and family has questions, concerns, or other issues**

**2. Summarizes**

**3. Clarifies future time when progress will again be discussed**

**4. Provides appropriate contact information if interim questions arise**

**5. Acknowledges patient and family, and closes interview**

- Did not use these behaviors
- Used 1 or 2 behaviors
- Used many behaviors, yet missed 1 or more opportunities
- Use these and other behaviors to consistently demonstrate this skill throughout

**8. Demonstrates empathy (includes the following):**

**1. Clinician’s demeanor is appropriate to the nature of the conversation**

**2. Shows compassion and concern**

**3. Identifies/labels/validates patient and family emotional responses**

**4. Responds appropriately to patient and family emotional cues**

- Did not use these behaviors
- Used 1 or 2 behaviors
- Used many behaviors, yet missed 1 or more opportunities
- Use these and other behaviors to consistently demonstrate this skill throughout

**9. Communicates accurate information (includes the following):**

**1. Accurately conveys the relative seriousness of patient's condition**

**2. Took other participating clinician’s input into account**

**3. Clearly conveys expected disease course**

**4. Clearly presents and explains options for future care**

**5. Gives enough clear information to enpower decision making**

- Did not use these behaviors
- Used 1 or 2 behaviors
- Used many behaviors, yet missed 1 or more opportunities
- Use these and other behaviors to consistently demonstrate this skill throughout

**What was this clinician best at?**

**10. Please select 3 choices:**

- Builds a relationship
- Opens the discussion
- Gathers information
- Understands patient's and family's perspective
- Shares information
- Reaches agreement
- Provides closure
- Demonstrates empathy
- Communicate accurate information

**11. Why did you choose those particular answers?**

**What could this clinician they improve on?**

**12. Please select 3 choices:**

- Builds a relationship
- Opens the discussion
- Gathers information
- Understands patient's and family's perspective
- Shares information
- Reaches agreement
- Provides closure
- Demonstrates empathy
- Communicate accurate information

**13. What could they have done better?**

***assessment for adapted from**

1. Calhoun AW, Rider EA, Meyer EC, Lamiani G, Truog RD. Assessment of communication skills and self-appraisal in the simulated environment: feasibility of multirater feedback with gap analysis. *Simulation in healthcare : journal of the Society for Simulation in Healthcare.* 2009;4(1):22-29.
