# Supplementary material for: Improving Child Neurology Residents' Communication Skills Through Objective Structured Clinical Exams
Source: MedEdPORTAL. 2021 Mar 4;17:11120. doi: 10.15766/mep_2374-8265.11120 (PMC7970633; doi:10.15766/mep_2374-8265.11120)
Supplement: Supplementary file 1 — Acute Stroke Scenario.docxMedical Error Scenario.docxStaring Spells Scenario.docxTourette Scenario.docxMigraine Scenario.docxDevelopmental Delay Scenario.docxDeath by Neurologic Criteria Scenario.docxPsychogenic Nonepileptic Events Scenario.docxNeonatal Hypoxic Ischemic Encephalopathy Scenario.docxFaculty & SP Assessment Form.docxLearner Self-Assessment Form.docxPost-OSCE Survey.docx [file mep_2374-8265.11120-s001.zip › L. Post-OSCE Survey.docx]

1. Overall, I felt the Child Neurology OSCE was beneficial to my education
   - Strongly disagree
   - Disagree
   - Neither agree nor disagree
   - Agree
   - Strongly agree
2. The information provided to me on the door instructions were clear and had enough information to approach the cases
   - Strongly disagree
   - Disagree
   - Neither agree nor disagree
   - Agree
   - Strongly agree
3. The overall expectations of me during the OSCE were clear
   - Strongly disagree
   - Disagree
   - Neither agree nor disagree
   - Agree
   - Strongly agree
4. I felt the case portrayals during the OSCE were realistic and believable
   - Strongly disagree
   - Disagree
   - Neither agree nor disagree
   - Agree
   - Strongly agree
5. I feel the feedback I received from the standardized patients (SPs) was helpful
   - Strongly disagree
   - Disagree
   - Neither agree nor disagree
   - Agree
   - Strongly agree
6. Please give specific feedback about the case ______, including the level of difficulty, what was most challenging about the case and how could the case be improved.
7. Please list 1-3 things that were most beneficial about the overall experience of the Child Neurology OSCE
8. Please lists 1-3 suggestions to improve the overall experience of the Child Neurology OSCE
