# Supplementary material for: Improving Child Neurology Residents' Communication Skills Through Objective Structured Clinical Exams
Source: MedEdPORTAL. 2021 Mar 4;17:11120. doi: 10.15766/mep_2374-8265.11120 (PMC7970633; doi:10.15766/mep_2374-8265.11120)
Supplement: Supplementary file 1 — Acute Stroke Scenario.docxMedical Error Scenario.docxStaring Spells Scenario.docxTourette Scenario.docxMigraine Scenario.docxDevelopmental Delay Scenario.docxDeath by Neurologic Criteria Scenario.docxPsychogenic Nonepileptic Events Scenario.docxNeonatal Hypoxic Ischemic Encephalopathy Scenario.docxFaculty & SP Assessment Form.docxLearner Self-Assessment Form.docxPost-OSCE Survey.docx [file mep_2374-8265.11120-s001.zip › _Educational_Summary_Report_11120.pdf]

MedEdPORTAL® The AAMC Journal of Teaching and Learning Resources

Original Publication Open Access

Improving Child Neurology Residents' Communication Skills Through Objective Structured Clinical Exams

Margie Ream, MD, PhD*, Dara V. F. Albert, DO, MEd, Todd Lash, MA, Nicole Verbeck, MPH, Pedro Weisleder, MD, PhD

*Corresponding author: dara.albert@nationwidechildrens.org

Abstract

Introduction: Child neurology has unique challenges in communication due to complex disorders with a wide array of prognoses and treatments. Effective communication is teachable through deliberate practice and coaching. Objective structured clinical exams (OSCEs) are one method of providing practice while assessing communication skills. Yet OSCEs have not been reported for child neurology residents. **Methods:** We developed simulated clinical cases centering on communication skills for child neurology residents, all with challenging clinical scenarios (e.g., disclosure of a medical error, psychogenic nonepileptic events). Standardized patients (SPs) portrayed the parents of pediatric patients and, in some scenarios, an adolescent patient. We used a modified Gap-Kalamazoo Communication Skills Assessment Form to assess communication skills. The assessment was completed by faculty, SPs, and the resident, and we measured agreement among raters. Residents were surveyed afterward regarding their experience. **Results:** Nine cases were developed and piloted. A total of 27 unique resident-case encounters with 16 individual trainees occurred over three annual implementations. Scores on the 360-degree assessment of communication skills showed that residents overwhelmingly underassessed their skills compared to other rater groups. Among 18 responses on the post-OSCE survey, the majority (77%) found the experience useful to their education and felt that the feedback from the SPs was helpful (61%) and the case portrayals were realistic (89%). **Discussion:** We implemented simulated cases for assessment and formative feedback on communication skills for child neurology residents. We provide a blueprint to develop this educational activity in other programs.

Keywords
Child Neurology, OSCE, Objective Structured Clinical Examination, Clinical Skills Assessment/OSCEs, Communication Skills, Neurology Education, Standardized Patient

Educational Objectives

By the end of this activity, learners will be able to:

1. Demonstrate the ability to provide reassurance to a patient and her or his parent(s) and gain acceptance of a difficult neurological diagnosis within a 30-minute encounter.
2. Demonstrate communication skills in what could be an adversarial situation with a challenging neurological diagnosis or a difference in goals of care between the provider and parents/patient.
3. Build on previously developed communication skills based on 360-degree feedback provided immediately following the clinical encounter.

Citation:
Ream M, Albert DVF, Lash T, Verbeck N, Weisleder P. Improving child neurology residents' communication skills through objective structured clinical exams. *MedEdPORTAL*. 2021;17:11120.
https://doi.org/10.15766/mep_2374-8265.11120

Introduction

Child neurology is a specialty with unique challenges in communication due to many complex disorders with a wide array of prognoses and treatments, such as epilepsy, cerebral palsy, stroke, and brain tumors. In addition, baseline knowledge of the nervous system is often lacking, especially among laypersons, and stigma still exists for patients with neurological disorders.[1-4] Moreover, many neurological disorders in children are progressive and without cure, leading to significant physical and mental impairments.[5] Therefore, child neurologists are often called to hold difficult conversations with patients and families in the hospital and outpatient settings. How medical information is conveyed to patients and families can significantly impact their ability to comprehend and cope with neurological disorders.[5]

Copyright © 2021 Ream et al. This is an open-access publication distributed under the terms of the Creative Commons Attribution license.

Consequently, effective communication is essential. As Storstein posited, “To communicate medical information that is certain to cause distress and sorrow, and yet convey empathy and support in the process, is one of the most difficult tasks in clinical practice.”[5] Child neurologists are faced with this difficult task on a regular basis.

Communication skills are essential skills that do not necessarily improve with experience.[6] They are highly valued by the Accreditation Council for Graduate Medical Education as core competencies.[7,8] Fortunately, these skills are teachable and are being taught at the medical school level.[5,6] However, there is limited literature regarding effective communication training during the child neurology residency.[5] One report of using simulated video-recorded consults for neurology trainees provides an example of using simulation to improve communication skills.[9] Another example of using simulation to teach neurology residents how to communicate a poor prognosis in the acute setting has been published in *MedEdPORTAL*.[10] In the oncology literature, there is evidence that practicing communication skills in a structured format allows for formative feedback that can help guide trainees and shape deliberate practice.[6] Furthermore, a recent review of the literature suggested that simulation-based medical education combined with deliberate practice may be superior to traditional education paradigms in achieving educational objectives.[11]

An objective structured clinical exam (OSCE) is a reliable, valid, and time-tested method of assessing trainees' communication skills as well as other abilities. The OSCE is a performance-assessment tool. It allows for observation of a resident's behavior in simulated but close-to-real-life scenarios. The merits of this type of assessment are its ability to give formative feedback on and coaching of behaviors that serve to facilitate deliberate practice. Deliberate practice is the intentional and sustained effort to improve proficiency in a skill or task. The driving force behind deliberate practice is the desire to achieve excellence. The goal is achieved through self-motivation, sustained practice, and willingness to accept feedback.[12] This assessment measures higher levels of Miller's pyramid, a well-accepted conceptual framework for clinical assessment: specifically, the “shows how” (performance) and “does” (action) levels.[13] OSCEs have been reported in adult neurology residency and in medical school education; however, they have not been reported for child neurology residents. We sought to develop an OSCE that could be used as a low-stakes formative assessment of communication skills in child neurology residents. We have published a report on this activity[14] and here describe its process so that others can replicate it.

Methods

We developed and piloted nine OSCE scenarios. Child neurology residents of all postgraduate years (PGY 3-PGY 5) participated. The activity was also open to adult neurology residents (PGY 2-PGY 4) and clinical neurophysiology fellows (PGY 6) interested in participating. The event took place at The Ohio State University College of Medicine's Clinical Skills Education and Assessment Center, which had full audiovisual recording capabilities. Standardized patients (SPs) were identified from a pool of trained and experienced SPs in the college's program. Most scenarios were written with an SP dyad in mind but could be performed by a single parent.

The nine clinical scenarios were developed by consensus of the program faculty, who were all practicing child neurologists and represented various career stages and subspecialty interests. The nine clinical scenarios we developed included acute stroke (case materials provided in Appendix A), disclosure of a medical error (case materials provided in Appendix B), staring spells (case materials provided in Appendix C), an adolescent with Tourette syndrome (case materials provided in Appendix D), an adolescent with migraine headaches (case materials provided in Appendix E), a child with developmental delay and negative diagnostic investigation (case materials provided in Appendix F), a child in the intensive care unit whose exam was compatible with the diagnosis of death by neurologic criteria (case materials provided in Appendix G), an adolescent with psychogenic nonepileptic events (case materials provided in Appendix H), and a neonate with severe hypoxic ischemic encephalopathy (case materials provided in Appendix I).

Basic Structure of the OSCE

The OSCE was repeated once per year over three consecutive academic years. Each OSCE contained three clinical case encounters with SPs portraying parents of a pediatric patient or an adolescent patient. Each case began with a clinical vignette describing the scenario. Instructions included counseling or discussion objectives to complete during the timed encounter with the SP. The case materials and door instructions for each case are provided in the appendices. The information in the door instructions was sufficient for the trainee to start the encounter from the point of education and counseling without requiring time for complete history taking and examination. Formative feedback focused on how the resident communicated rather than what the resident communicated. This situation resembled

Copyright © 2021 Ream et al. This is an open-access publication distributed under the terms of the Creative Commons Attribution license.

real-life clinical encounters during which even experienced neurologists might not have all the answers but still need to discuss diagnostic and/or management possibilities with a family. Cases had varying levels of complexity and difficulty. Residents of all levels encountered the same three cases during each OSCE. Each resident rotated through three 40-minute stations, which included the 20-minute encounter followed by a 20-minute feedback session with the SPs. Direct verbal feedback from the SPs after each encounter was the first element of feedback provided to each learner.

SP Training

An experienced SP trainer (Todd Lash) helped to identify SPs who routinely worked at the clinical skills laboratory for medical student assessment. SPs received training materials for portrayals at least 1 week in advance. The assessment forms used by the SPs are included in the Appendix J. Just prior to each OSCE event, we met with the SPs to discuss nuances of the cases and help recalibrate the SPs' expectations from medical students to residents.

Learner Assessment and Feedback

We used the Gap-Kalamazoo Communication Skills Assessment Form, a published instrument used in medical education to assess communication skills.[15,16] We modified the response options from a qualitative Likert-type scale to four behaviorally anchored options—*did not use these behaviors*; *used one or two behaviors*; *used many behaviors, yet missed one or more opportunities*; and *used these and other behaviors to consistently demonstrate this skill throughout*—after receiving feedback from faculty. The modified Gap-Kalamazoo Communication Skills Assessment Form used for learner self-assessment is available in Appendix K; the faculty and SP assessment form is available in Appendix J. We aimed to increase rater consistency and interrater reliability by reducing subjectivity of the scoring options. Prior to the OSCE, all faculty and SPs were trained on how to use the modified Gap-Kalamazoo to minimize construct-irrelevant variance. Following the encounter, faculty, SP(s), and the resident completed the modified Gap-Kalamazoo electronically, resulting in a 360-degree assessment of communication skills. If the case was portrayed by two SPs, they completed one form together. In addition, after each encounter, SPs provided unstructured verbal feedback to the trainees.

Although each clinical scenario was different, all assessed communication skills and were scored on the same assessment form. The number of cases allowed us to better sample communication skills and decrease construct underrepresentation as a threat to validity. We did not go through the process of standard setting to establish a pass/fail line as this activity was not used as a summative assessment. Trainees were not asked to prepare prior to the OSCE events. They were not given the assessment form but were informed of the logistics of the event and that communication skills would be assessed.

Encounters were video and audio recorded in the clinical skills laboratory. Each encounter took place in a room akin to a clinic room. A faculty member observed behind a one-way mirror and could listen to the audio through headphones. Feedback was provided in verbal and written formats. Directly after each encounter, SPs provided verbal feedback to each trainee. Two forms of written feedback were subsequently provided for each case—the modified Gap-Kalamazoo completed by the observing faculty and the modified Gap-Kalamazoo completed by the SP(s). Residents also had access to their recorded encounters. After receiving written feedback, the trainees were asked to watch their performance in the recorded encounter and reflect on what they found most challenging. Trainees then participated in a group debriefing session during which they reflected on the experience and good communication skills were reinforced. Program leadership (Dara V. F. Albert and Margie Ream) facilitated these sessions and provided advice from their own experiences with difficult conversations in practice. In order to understand the participants' reactions to the program and its components, we asked residents to complete a post-OSCE survey (Appendix L).

Statistical Analysis

We compared rating scores between faculty, SP, and resident self-ratings. Percentages of agreement between raters were calculated for each of the clinical scenarios. We used descriptive statistics to analyze the Gap-Kalamazoo scores and the responses to the post-OSCE survey. Interrater reliability analyses could not be performed given the small numbers of participants and raters. More details about rater agreement and disagreement are included in our other publication describing this project.[14]

Results

We implemented a child neurology–focused communication OSCE and piloted all nine cases over three academic years. A total of 27 unique resident-case encounters with 16 individual trainees occurred over the three implementations. These included four adult neurology residents and two clinical neurophysiology fellows who volunteered to participate (adult and child neurology trainees from PGY 2 through PGY 6 participated). Three residents experienced all nine cases. All

Copyright © 2021 Ream et al. This is an open-access publication distributed under the terms of the Creative Commons Attribution license.

cases were rated by a faculty observer, resident, or fellow, as well as by the SPs, for a total of three completed assessment forms per encounter. Average total scores for each rater type are shown in Table 1. We did see some instances of complete agreement between raters, but that was infrequent. The frequencies and percentages of agreement and disagreement for each item are shown in Table 2.

Resident Feedback

We received 18 responses on the post-OSCE survey (67% of participants). The majority (77%) found the experience useful to their education, assessed the feedback from the SPs as helpful (61%), and thought that the case portrayals were realistic (89%). Nearly unanimously, the residents reported that the most valuable part of the experience was the real-time and direct feedback from the patients/parents. This was a unique opportunity, as trainees rarely get this type of feedback from true patients and families. One quote from the open-response questions on the survey was the following:

> What a rare but helpful opportunity to receive direct feedback during patient encounters in a formalized manner. This should be a much more emphasized part during education as medical knowledge can be obtained by self-study; patient interaction obviously relies on a different set of skills and parameters. Reflection of cases in this environment allows to focus on important aspects in delivery of health care.

Seven learners either were neutral or disagreed that feedback from the SPs was helpful. Two who responded neutral and two who disagreed with this question did not provide comments in the free text to explain their responses. The remaining learners who disagreed commented that the feedback was “somewhat surface level and rudimentary,” that we needed to “train SPs a little more on what types of feedback are helpful,” and that “the SP for the teenage kid case came across as rude while giving feedback. It almost felt like they were scolding me.” Responses to the survey were anonymous, so those who disagreed with any questions could not be directly probed for more information.

Table 1. Average Total Scores for Residents, SPs, and Faculty Raters for Each OSCE and Across All Three OSCE Events

Year	Resident	SP	Faculty
OSCE year 1	75%	83%	81%
OSCE year 2	68%	76%	82%
OSCE year 3	75%	86%	82%
Average	72%	82%	82%

Abbreviations: OSCE, objective structured clinical exam; SP, standardized patient.

Table 2. Agreement Between Raters on the Different Assessment Items

	No. (%)	
Item	Faculty and Standardized Patient Agreement	Trainee and Faculty Agreement
Builds a relationship	42 (47)	40 (45)
Opens the discussion	44 (49)	25 (28)
Gathers information	36 (40)	34 (38)
Understands patient’s perspective	30 (34)	32 (36)
Shares information	49 (55)	30 (34)
Reaches agreement	37 (42)	37 (42)
Provides closure	40 (45)	38 (43)
Demonstrates empathy	50 (56)	34 (38)
Communicates accurate information	41 (46)	34 (38)

We have included a Word version of the post-OSCE survey as Appendix L.

Discussion

We have described the development and implementation of an OSCE for child neurology residents focusing on communication skills. At the core of the activity was the unique opportunity to receive 360-degree formative feedback from SPs, faculty raters, peers, and introspection. The breadth of common but challenging clinical scenarios mirrored pediatric neurology practice and allowed for multiple opportunities to paint a more valid picture of communications skills.

Limitations

Our project had several limitations, some of which may limit its generalizability to other programs and pose threats to validity of the activity as an assessment. The first of these was the small number of trainees who underwent a limited number of cases in the activity. Although all the cases sampled the same domain of communication skills and used the same assessment form, each case was unique in its content and challenges. The cases were internally developed by consensus of experts and were meant to be a representative sample of clinical scenarios. However, these scenarios did not represent every type of case that trainees could encounter; therefore, there was risk of construct underrepresentation. There was also the possibility of construct-irrelevant variance in the content of the cases, case difficulty, and case portrayals from the SPs. We recognize that in real life, residents may not be faced with these scenarios without a supervising physician present. However, this OSCE allowed us to challenge the residents’ comfort level in a way that real-life practice in training does not. Furthermore, the small number of raters per case limited the ability to calculate interrater reliability. In addition, we did receive feedback from some participants that more clinical detail in the door instructions would have been helpful, particularly for trainees with less experience.

Copyright © 2021 Ream et al. This is an open-access publication distributed under the terms of the Creative Commons Attribution license.

Of importance, we chose to modify the Gap-Kalamazoo Communication Skills Assessment Form based on feedback from our faculty. This modification made the form an unvalidated instrument, and further study would be required to provide assess its validity. The modifications did, however, make the form easier for our faculty to complete. If programs wishing to implement this OSCE prefer to use a validated instrument, the unmodified version could be used.[17]

Advice for Implementation

We would like to share some recommendations for other programs that wish to develop and implement this activity. We recommend piloting the cases to ensure the events run smoothly.

Training the SPs on the nuances of each scenario is of the utmost importance. Specifically, role-playing with the SPs prior to the event so that they had a good understanding of the conditions at hand was something we found very helpful. In addition, organizers should provide guidance to the SPs on the kind of feedback that would be beneficial to a trainee at the graduate level of medical education. This is especially important for SPs who typically work with medical students. SPs should be trained on the assessment form used to assess the trainees' performance so that they can provide reliable written feedback. We recommend working with an SP trainer who has experience with medical case portrayal.

Faculty observers also need to be trained on the assessment form. We suggest a group-training session to help develop a shared mental model and increase consistency across raters. If feasible, we suggest the presence of faculty who can provide onsite live rating of the encounters. Although our residents reported that feedback from the SPs was the most valuable, they also valued feedback from faculty. This phenomenon has previously been reported.[18,19] Some authors have highlighted that there may be discrepancies between real-time and video observations. That difference could impact pass/fail determinations if the assessment is summative.[20] Also, remote video rating depends on high-quality audiovisual recording and remote streaming capabilities. By definition, asynchronous and remote rating delays feedback to residents. This issue can result in the loss of nuanced advice. The Gap-Kalamazoo is a useful instrument for measuring communication skills across a wide variety of medical encounters. However, the instrument is not specific to communication in neurology. Given the unique challenges we face in clinical practice, it would be beneficial to developed and validate an assessment tool more specific to our specialty. Lastly, it may be valuable to consider including the opportunity to watch an expert faculty member engage in one of these difficult simulated encounters as an exemplar encounter. Another option might be to consider highlighting examples of good communication from peers.

Conclusions

We developed nine standardized cases to simulate difficult conversations encountered in child neurology practice. These cases afforded an opportunity for trainees to practice communication skills and receive feedback. This activity was well received by our trainees, with the most valuable piece being the feedback they received from the patients' perspective, something they would not be able to get in real-life scenarios.

Appendices

A. Acute Stroke Scenario.docx
B. Medical Error Scenario.docx
C. Staring Spells Scenario.docx
D. Tourette Scenario.docx
E. Migraine Scenario.docx
F. Developmental Delay Scenario.docx
G. Death by Neurologic Criteria Scenario.docx
H. Psychogenic Nonepileptic Events Scenario.docx
I. Neonatal Hypoxic Ischemic Encephalopathy Scenario.docx
J. Faculty & SP Assessment Form.docx
K. Learner Self-Assessment Form.docx
L. Post-OSCE Survey.docx

All appendices are peer reviewed as integral parts of the Original Publication.

Margie Ream, MD, PhD: Assistant Professor, Department of Pediatrics, Division of Child Neurology, Nationwide Children's Hospital and The Ohio State University College of Medicine

Dara V. F. Albert, DO, MEd: Assistant Professor, Department of Pediatrics, Division of Child Neurology, Nationwide Children's Hospital and The Ohio State University College of Medicine; ORCID: https://orcid.org/0000-0002-3867-2297

Todd Lash, MA: Education Resource Specialist, Clinical Skills Education and Assessment Center, The Ohio State University College of Medicine

Nicole Verbeck, MPH: Research Specialist, Office of Curriculum and Scholarship, The Ohio State University College of Medicine

Pedro Weisleder, MD, PhD: Professor, Department of Pediatrics, Division of Child Neurology, Nationwide Children's Hospital and The Ohio State University College of Medicine

Copyright © 2021 Ream et al. This is an open-access publication distributed under the terms of the Creative Commons Attribution license.

Acknowledgments
We would like to acknowledge the talented actors who portrayed the patients and parents in our clinical scenarios and provided valuable feedback to our trainees. We would also like to acknowledge the residents and fellows who participated in this pilot project and provided their feedback regarding the program.

Disclosures
None to report.

Funding/Support
This project was funded by the American Board of Psychiatry and Neurology Innovation in Education Award.

Ethical Approval
Reported as not applicable.

References

1. Demirci S, Dönmez CM, Gündoğar D, Baydar ÇL. Public awareness of, attitudes toward, and understanding of epilepsy in Isparta, Turkey. *Epilepsy Behav*. 2007;11(3):427-433. https://doi.org/10.1016/j.yebeh.2007.08.005
2. Jacoby A, Gorry J, Gamble C, Baker GA. Public knowledge, private grief: a study of public attitudes to epilepsy in the United Kingdom and implications for stigma. *Epilepsia*. 2004;45(11):1405-1415. https://doi.org/10.1111/j.0013-9580.2004.02904.x
3. Jacoby A, Snape D, Baker GA. Epilepsy and social identity: the stigma of a chronic neurological disorder. *Lancet Neurol*. 2005;4(3):171-178.
4. Patel V, Chisholm D, Parikh R, et al.; DCP MNS Authors Group. Global priorities for addressing the burden of mental, neurological, and substance use disorders. In: Patel V, Chisholm D, Dua T, Laxminarayan R, Medina-Mora ME, eds. *Mental, Neurological, and Substance Use Disorders*. 3rd ed. International Bank for Reconstruction and Development/World Bank; 2015:1-27. Jamison DT, Nugent R, Gelband H, et al., eds. *Disease Control Priorities*; vol. 4.
5. Storstein A. Communication and neurology—bad news and how to break them. *Acta Neurol Scand*. 2011;124(s191):5-11. https://doi.org/10.1111/j.1600-0404.2011.01550.x
6. Arnold SJ, Koczwara B. Breaking bad news: learning through experience. *J Clin Oncol*. 2006;24(31):5098-5100. https://doi.org/10.1200/JCO.2006.08.6355
7. Jardine D, Deslauriers J, Kamran SC, Khan N, Hamstra S, Edgar L. *Milestones Guidebook for Residents and Fellows*. Accreditation Council for Graduate Medical Education; 2017.
8. Batalden P, Leach D, Swing S, Dreyfus H, Dreyfus S. General competencies and accreditation in graduate medical education. *Health Aff (Millwood)*. 2002;21(5):103-111. https://doi.org/10.1377/hlthaff.21.5.103
9. Smith PEM, Fuller GN, Kinnersley P, Brigley S, Elwyn G. Using simulated consultations to develop communications skills for neurology trainees. *Eur J Neurol*. 2002;9(1):83-87. https://doi.org/10.1046/j.1468-1331.2002.00339.x
10. Smyth P, Sim V. Objective structured clinical examination (OSCE) station on communicating poor prognosis to the family in a neurological acute care setting. *MedEdPORTAL*. 2014;10:9700. https://doi.org/10.15766/mep_2374-8265.9700
11. McGaghie WC, Issenberg SB, Cohen ER, Barsuk JH, Wayne DB. Does simulation-based medical education with deliberate practice yield better results than traditional clinical education? A meta-analytic comparative review of the evidence. *Acad Med*. 2011;86(6):706-711. https://doi.org/10.1097/ACM.0b013e318217e119
12. Ericsson KA. Deliberate practice and acquisition of expert performance: a general overview. *Acad Emerg Med*. 2008; 15(11):988-994. https://doi.org/10.1111/j.1553-2712.2008.00227.x
13. Miller GE. The assessment of clinical skills/competence/ performance. *Acad Med.* 1990;65(9):S63-S67. https://doi.org/10.1097/00001888-199009000-00045
14. Albert DVF, Ream M, Verbeck N, Lash T, Weisleder P. An objective structured clinical examination of communication skills for child neurology residents. *Pediatr Neurol*. 2021;114:68-74. https://doi.org/10.1016/j.pediatrneurol.2020.09.004
15. Joyce BL, Steenbergh T, Scher E. Use of the Kalamazoo Essential Elements Communication Checklist (Adapted) in an institutional interpersonal and communication skills curriculum. *J Grad Med Educ*. 2010;2(2):165-169. https://doi.org/10.4300/JGME-D-10-00024.1
16. Porcerelli JH, Brennan S, Carty J, Ziadni M, Markova T. Resident ratings of communication skills using the Kalamazoo Adapted Checklist. *J Grad Med Educ*. 2015;7(3):458-461. https://doi.org/10.4300/JGME-D-14-00422.1
17. Calhoun AW, Rider EA, Meyer EC, Lamiani G, Truog RD. Assessment of communication skills and self-appraisal in the simulated environment: feasibility of multirater feedback with gap analysis. *Simul Healthc*. 2009;4(1):22-29. https://doi.org/10.1097/SIH.0b013e318184377a
18. Chen TC, Lin MC, Chiang YC, Monrouxe L, Chien SJ. Remote and onsite scoring of OSCEs using generalisability theory: a three-year cohort study. *Med Teach*. 2019;41(5):578-583. https://doi.org/10.1080/0142159X.2018.1508828
19. Chan J, Humphrey-Murto S, Pugh DM, Su C, Wood T. The objective structured clinical examination: can physician-examiners participate from a distance? *Med Educ*. 2014;48(4):441-450. https://doi.org/10.1111/medu.12326
20. Sturpe DA, Huynh D, Haines ST. Scoring objective structured clinical examinations using video monitors or video recordings. *Am J Pharm Educ*. 2010;74(3):44. https://doi.org/10.5688/aj740344

Received: June 20, 2020
Accepted: December 30, 2020
Published: March 4, 2021

Copyright © 2021 Ream et al. This is an open-access publication distributed under the terms of the Creative Commons Attribution license.
